# Active site remodelling of a cyclodipeptide synthase redefines substrate scope

**DOI:** 10.1038/s42004-022-00715-2

**Published:** 2022-08-25

**Authors:** Emmajay Sutherland, Christopher John Harding, Clarissa Melo Czekster

**Affiliations:** School of Biology, Biomedical Sciences Research Complex, University of St Andrews, St Andrews, Fife UK

**Keywords:** Ligases, X-ray crystallography, Biocatalysis

## Abstract

Cyclodipeptide synthases (CDPSs) generate a wide range of cyclic dipeptides using aminoacylated tRNAs as substrates. Histidine-containing cyclic dipeptides have important biological activities as anticancer and neuroprotective molecules. Out of the 120 experimentally validated CDPS members, only two are known to accept histidine as a substrate yielding cyclo(His-Phe) and cyclo(His-Pro) as products. It is not fully understood how CDPSs select their substrates, and we must rely on bioprospecting to find new enzymes and novel bioactive cyclic dipeptides. Here, we developed an in vitro system to generate an extensive library of molecules using canonical and non-canonical amino acids as substrates, expanding the chemical space of histidine-containing cyclic dipeptide analogues. To investigate substrate selection we determined the structure of a cyclo(His-Pro)-producing CDPS. Three consecutive generations harbouring single, double and triple residue substitutions elucidated the histidine selection mechanism. Moreover, substrate selection was redefined, yielding enzyme variants that became capable of utilising phenylalanine and leucine. Our work successfully engineered a CDPS to yield different products, paving the way to direct the promiscuity of these enzymes to produce molecules of our choosing.

## Abstract (web summary)

Cyclodipeptide synthases (CDPSs) generate a wide range of cyclic dipeptides using aminoacylated tRNAs as substrates, however the substrate selection mechanism is not yet known. Here, the authors investigate the substrate promiscuity of two histidine-incorporating CDPSs to generate an extensive library of products which complement the chemical realm of histidine-containing cyclic dipeptides.

## Introduction

Cyclodipeptide synthases (CDPSs) use aminoacylated tRNA (aa-tRNA) substrates to form peptide bonds between two amino acids yielding a cyclic dipeptide product (CDP)^1^. CDPs contain a diketopiperazine ring, a scaffold that has been coined as privileged due to remarkable properties such as proteolytic resistance, blood-brain barrier permeability and the ability to mimic functional pharmacophores^2–4^. CDPSs act in combination with tailoring enzymes, adding significant complexity to the types of natural products that can be produced^5,6^. Because computational prediction of the specificity of CDPSs is challenging^7^, the determination of substrates and products of each enzyme requires experimental testing, in a time consuming and low throughput process^8–10^. Prior to the work reported herein, there were no reports of successfully engineering CDPS enzymes to direct substrate selection.

**Fig. 1:**
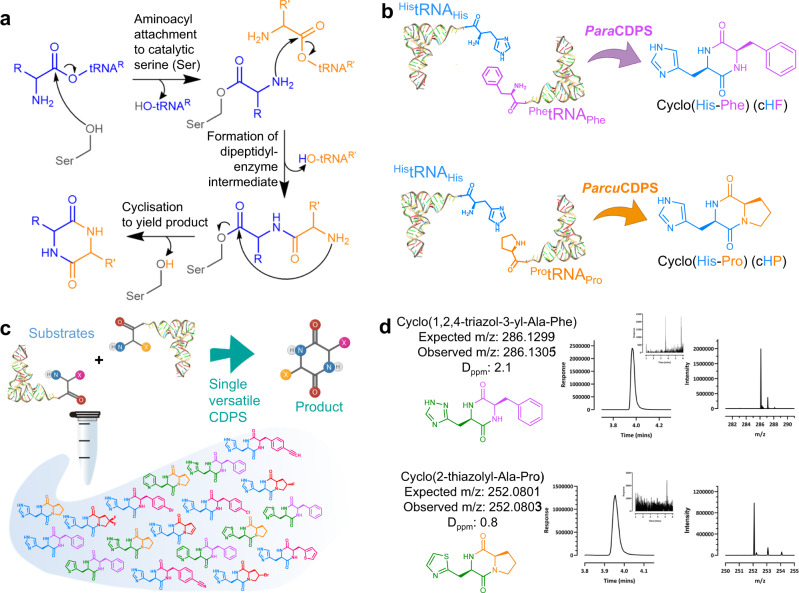
CDPS mechanism and reaction and products. **a** General mechanism of CDPS enzymes as elucidated from AlbC^12–17^. **b** Enzymes characterised here, both use aminoacylated tRNAs as substrates to produce a cyclic dipeptide. *Para*CDPS from *Parabacteroides sp. 20_3* (GenBank: EFK64745.1) produces cyclo(L-His-L-Phe), and *Parcu*CDPS from *Parcubacteria bacterium RAAC4_OD1_1* (GenBank: ETB63777.1) can synthesise both cyclo(L-His-L-Glu) – not shown – and cyclo(L-His-L-Pro). **c** Strategy employed here, using a single enzyme catalyst to produce several cyclodipeptide products; **d** Representative LC-HRMS spectra showing production of new molecules. The extracted ion chromatogram (EIC) for each is shown with the corresponding mass spectra from each peak. The inset shows an EIC from a control lacking CDPS. Spectra for all cyclodipeptides described here are on SI Figs. 7 and 8.

AlbC from *Streptomyces noursei* was the first CDPS to be discovered in 2002^11^. Since then, there have been 120 CDPSs with empirically determined function and more than 1000 predicted members of the family (Uniprot: IPRO38622). CDPSs are known for their promiscuity as most are capable of naturally synthesising more than one product^8^. This interesting characteristic is due to two separate solvent accessible pockets (referred to as P1 and P2) which interact with the specific aminoacylated tRNA (aa-tRNA) required for product formation^12^. Extensive investigation into the mechanism using both experimental and computational methods elucidated the catalytic mechanism of CDPSs^12–14^. The reaction proceeds via a ping pong mechanism, the first half reaction in P1 forms an aminoacyl-enzyme intermediate covalently bound to the catalytic serine (S26 in AlbC)^15^. The second step forms a dipeptidyl-enzyme intermediate which undergoes cyclisation facilitated by a conserved tyrosine residue (Fig. 1a)^14^. This reaction relies heavily on four known residues which are conserved throughout the family: S26, Y167, E171 and Y189 (numbering respective to AlbC). Further research into CDPSs highlighted that the family could be characterised into two sub-groups based on the residues present on P1/P2. The larger of the two families, NYH, features N40, Y202 and H203 whilst XYP has X40 where X is non-conserved, Y202 and P203^16^. Crystal structures from representatives of both families are available: AlbC; Rv2275; YvmC^17^, and BtCDPS^18^ from NYH sub-family and *Nbra*-CDPS, *Rgry*-CDPS, *Fdum*-CDPS from XYP^16^.

Here, we focus on the only two known CDPS enzymes which synthesise bioactive histidine-containing cyclic dipeptides (Fig. 1b)^19–23^. Cyclo(L-His-L-Phe) (cHF) encompasses the backbone structure of the anti-tumour compound plinabulin, while cyclo(L-His-L-Pro) (cHP) is endogenous to the human body and proposed as a neuroprotective peptide against Parkinson’s disease and amyotrophic lateral sclerosis^24,25^, as well as a molecule involved in the gut-brain-axis crosstalk^26^, with effects on glucose metabolism^27^. Thus, it would be advantageous to expand the chemical space of histidine-containing cyclic dipeptide analogues we could produce by using CDPS enzymes.

We describe a facile strategy for cyclic dipeptide production with superior yield and decreased cost/labour. Using canonical and non-canonical amino acids as substrates, we generated a diverse library of unprecedented compounds (Supplementary Table 1 and 2). We then used small molecule substrates as chemical probes to characterise substrate binding pockets P1, which is more stringent and deeper, and P2, which is shallower and more solvent exposed^18^. To investigate histidine recognition on P1, we solved crystal structures of the cyclo(His-Pro)-producing enzyme, as well as several mutants. We rationally engineered P1 to become more hydrophobic and deeper, steering the substrate specificity away from histidine and towards more hydrophobic amino acids. These mutants displayed a remarkable shift in substrate specificity from the previously accepted histidine to two new substrates – leucine and phenylalanine. Therefore, our CDPS variants highlighted residues in P1 which are key for the recognition of histidine, unveiling important characteristics of how CDPSs select polar substrates. This has wide implications in our capacity to predict function as well as engineer CDPS enzymes to produce molecules of our choosing.

## Results and discussion

### Enzymes that use histidinyl-tRNA as substrate

*Para*CDPS from *Parabacteroides sp. 20_3* (GenBank: EFK64745.1) produces cyclo(His-Phe)^8^ whilst *Parcu*CDPS from *Parcubacteria bacterium RAAC4_OD1_1* (GenBank: ETB63777.1) can synthesise both cyclo(His-Glu) and cyclo(His-Pro)^9^. We produced both enzymes in high yield, protein purity and identity were confirmed by intact protein mass spectrometry and SDS-PAGE analysis and their activity and reaction products were verified using purified components in vitro (Supplementary Fig. 1).

### Facile production of tRNA simplifies CDP synthesis

CDPS enzymes use aa-tRNA molecules as substrates, hijacking the already aminoacylated tRNA within cells for cyclodipeptide synthesis^10^. Purified tRNA was previously synthesised by us and others using a time consuming in vitro transcription reaction which included an initial PCR of the desired tRNA sequence to amplify the DNA template encoding the desired tRNA sequence, followed by in vitro transcription using a mutant T7 RNA polymerase (Δ172-173) to ensure homogeneous 3’-end in the tRNA, finishing with a phenol-chloroform extraction to yield purified tRNA (Supplementary Fig. 3)^28^. This method, whilst reliable, is costly, lengthy and requires specialised materials.

**Fig. 2:**
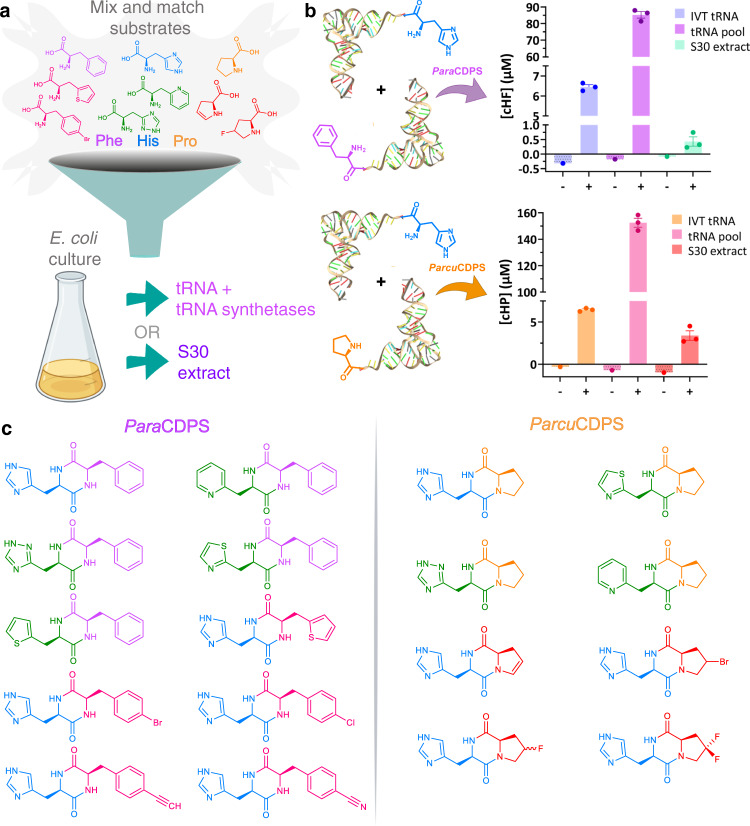
Production of novel cyclodipeptides using *Para*CDPS and *Parcu*CDPS. **a** Cartoon schematic depicting possible substrates for each CDPS studied herein. The tRNA structure of the aminoacylated tRNA is from the PDB – 1EHZ. **b** Quantification of CDP production by LC-MS using a standard calibration curve. Data is shown as single point in triplicate with error bars plotting the standard error of the mean (SEM). Measurements were taken from distinct samples. The presence (+) and absence (−) of CDPS is displayed for each source of tRNA: in vitro transcribed (IVT) tRNA; tRNA pool and S30 extract. It is evident that the tRNA pool is a far superior method of producing CDP for both CDPSs. **c** Generation of a cyclic dipeptide library using *Para*CDPS and *Parcu*CDPS using non-canonical amino acids: histidine derivatives are shown in green; phenylalanine derivatives in pink and proline derivatives in red.

Aiming to bypass the individual purification of the amino acid tRNA synthetases as well as the individual tRNAs, we tested a bacterial lysate (S30 extract) isolated from *E. coli* containing amino acids, tRNA and aminoacyl-tRNA synthetases^29^. Although initially easy to produce, this S30 extract was not as efficient at yielding CDPs as expected (Fig. 2b). Furthermore, use of non-canonical amino acids with the S30 extract was unsuccessful due to the high levels of endogenous aminoacylated tRNA present.

We then adapted a procedure by Mechulam *et al*. which performed a liquid-liquid organic extraction of total tRNA, followed by subsequent clean up to produce a ‘pool’ of tRNA naturally found in *E. coli*^30^. Using this method, a highly concentrated stock of all the tRNAs required for our experiments was purified and used with any CDPS/aaRSs (aminoacyl-tRNA synthetase) combination of choice. More specifically, *Para*CDPS and *Parcu*CDPS could use this tRNA pool in conjunction with purified aminoacyl-tRNA synthetases to generate their respective products – cHF and cHP – in relatively high yield (Fig. 2b). In comparison, we also performed reactions using in vitro transcribed tRNA to confirm that the same cyclodipeptides were produced, however the tRNA pool method remains significantly cheaper, easier, and faster than previously described methods and has become a staple in our research with CDPSs. This method could be used for other enzymes that use aa-tRNAs as substrates, such as Fem transferases and LanB-like dehydratases^31^. Overall, the tRNA pool was a far superior alternative to produce high quality tRNA for use and therefore was employed in all subsequent reactions.

### Incorporation of non-canonical amino acids into cyclic dipeptides

Recently, a review was published detailing non-canonical amino acids known to be accepted by aminoacyl-tRNA synthetases from *E.coli*, summarizing extensive research in this field by several labs worldwide^32^. Additionally, the use of non-canonical substrates with CDPSs was previously carried out employing in vivo experiments exclusively^33^. Consequently we explored the capability of *Para*CDPS and *Parcu*CDPS in accepting unnatural amino acid analogues within our in vitro experimental setup as described previously (reaction details found in SI). Using a range of commercially available non-canonical analogues with the tRNA pool and respective aa-tRNA synthetases (Fig. 2a), a library of diverse CDPs was produced from just two CDPSs (Fig. 2c). We imposed strict allowances on the mass deviation to confidently assess if an unnatural amino acid was accepted. All experiments were carried out using a high resolution mass spectrometer so only unique peaks (in comparison to controls) with area >100 and observed masses under 5 ppm from the predicted exact mass were taken into consideration for a product to be deemed ‘present’. *Para*CDPS and *Parcu*CDPS both accepted the same histidine analogues - H-β-(2-Thiazolyl)-DL-alanine and 3-(2-pyridyl)-L-alanine—with *Parcu*CDPS also able to use β-(1,2,4-Triazol-3-yl)-DL-alanine. Since all analogues employed were previously shown to be substrates for histidinyl tRNA synthetase (HisRS)^34^, the CDPS enzyme could be posing as an additional sieve for substrate selection. Prior to our work, no information was available about histidine recognition by CDPSs. The unnatural substrate utilisation emphasises that the nitrogen on position three of the imidazole ring is important for substrate recognition. The incorporation of the pyridyl ring indicates that this enzyme can accept larger ring structures however only one isomer (2,3-pyridyl)-L-alanine was found to be introduced into a CDP. Moreover, the rejection of isomers 3-(3-) and 3-(4-pyridyl)-L-alanine supports the observation that a nitrogen may be required in close proximity to the alpha carbon of the amino acid. Supplementary Note 1 and Supplementary Tables 1 and 2 summarize all analogues tested, Supplementary Table 5 in Supplementary Note 9 details the novel cyclic dipeptides produced here and Supplementary Figs. 7-10 include LC-MS chromatograms of the cyclic products.

To further expand our diketopiperazine library we exploited the increased promiscuity of CDPSs to produce cHF analogues using a phenylalanyl tRNA synthetase mutant (PheRS-A294G) which has a wider binding pocket allowing interactions with a larger range of related Phe analogues^35^. All but two of the known non-canonical amino acids previously shown to be accepted by PheRS-A294G were incorporated into the ring by *Para*CDPS (Fig. 2c). All halogen para-substitutions in the phenyl ring were easily accepted by the CDPS, but not para substitutions introducing polar groups such as amine, nitro and azido (Supplementary Table 1). To produce cHP variants, several halogenated proline analogues reported as substrates for ProRS were also utilised by *Parcu*CDPS. 4-Bromo-L-proline was not previously shown to be a substrate for ProRS but it was hypothesised to display similar chemistry as 4-fluoro-L-proline^32^. *Parcu*CDPS can tolerate conservative derivatisations of proline however functional groups including hydroxyl and amines on the ring were not accepted. This may be due to positioning in the pocket causing steric clashes and forcing the amino acid into unfavourable conformations for cyclisation. Although *Parcu*CDPS also produces cHE (Supplementary Note 2 and Supplementary Fig. 11), it did not accept any glutamate analogues, demonstrating surprisingly narrow specificity.

### CDP formation using a minimal substrate

Previous research from our group demonstrated the use of small molecules containing a dinitrobenzyl ester coupled to an amino acid (aa-DBE) as substrates for CDPSs^18^. These minimal substrates are useful tools to investigate substrate specificity, and binding to P1/P2, while also presenting an alternative to using aa-tRNA as substrates. Previously, the use of a shortened tRNA was employed in combination with aa-tRNAs on the CDPS from *Nocardia brasiliensis*^36^, but the hybrid reaction conditions detailed herein use a unique combination of aa-DBE and aa-tRNA as substrates which has not previously been published.

**Fig. 3:**
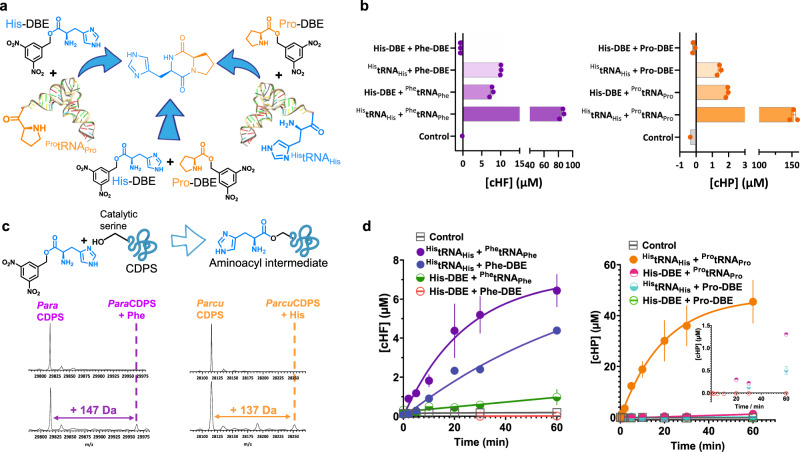
Use of minimal substrates to yield CDPs. **a** Reaction scheme highlighting the three possible combinations when using DBE substrates in conjunction with aa-tRNA. **b** Quantification of product yield from aa-DBE and aa-tRNA reactions with each CDPS. Reactions with *Para*CDPS are shown in purple on the left and reactions with *Parcu*CDPS are in orange on the right. **c** Reaction scheme showing covalent attachment of amino acid to catalytic serine of CDPS if accepted in P1 and intact protein mass spectrometry of trapped acyl-enzyme intermediates for *Para*CDPS and *Parcu*CDPS. Contrary to our original hypothesis, *Para*CDPS binds phenylalanine in P1 as shown by the mass relating to *Para*CDPS + Phe - *Parcu*CDPS, however, binds histidine in the first pocket. **d** Time course assay for product formation of cHF and cHP using *Para*CDPS and *Parcu*CDPS respectively. Lines are fit to an exponential equation to obtain rates of formation as follows: His-tRNA + Phe-tRNA = 0.0024 min-1; His-tRNA + Phe-DBE = 0.0048 min-1; His-DBE + Phe-tRNA = 0.0022 min-1; His-DBE + Phe-DBE = too low to accurately fit; His-tRNA + Pro-tRNA = 0.05 min-1; His-DBE + Pro-tRNA = 0.00002 min-1; His-tRNA + Pro-DBE and His-DBE + Pro-DBE were too low to accurately fit. Inset shows a very small amount of product is formed when one aa-DBE substrate is used but not in the reaction with two aa-DBE substrates.

To test if hybrid reaction conditions would yield product, we synthesised His-DBE; Pro-DBE, and Phe-DBE (Supplementary Figs. 4 and 5). Reactions containing only minimal aa-DBE substrates were highly unfavourable and yielded very little CDP. In contrast, hybrid reactions using all available combinations for aa-DBE and aa-tRNA substrates (Fig. 3a) revealed that more product was formed using a combination of the aa-tRNA/aa-DBE substrates, albeit with lower overall yield when compared to the natural tRNA substrates (Fig. 3b). It is important to note that aa-DBE has a limited half-life, which also impacts reaction yields^18^. In these hybrid reaction conditions, we hypothesised that the small aa-DBE substrate would be turned over to generate a cyclic dipeptide product when occupying the deeper narrower P1 pocket, while P2 was occupied by an aa-tRNA, and not the other way around since aa-DBE occupying P2 would result in unproductive binding conformations, leading to aa-DBE hydrolysis and no cyclic dipeptide formed.

Our results show that *Para*CDPS generated more product using ^His^tRNA_His_ **+** Phe-DBE, in disagreement to what would be expected if histidine was occupying P1 given the hypothesis above was correct. Consequently, we investigated both enzymes using a trapped acyl-enzyme intermediate experiment developed in-house, which exploits the minimal aa-DBE substrate as a chemical probe to verify substrate binding order. Here we saw that *Para*CDPS accepts Phe in P1 instead of His whereas *Parcu*CDPS accepts His in P1 (Fig. 3c). This, therefore, corroborates our hypothesis, and most likely aa-DBE occupying P2 can sample several unproductive conformations leading to DBE hydrolysis before successful product formation.

Minimal aa-DBE substrates are also useful tools to investigate kinetics of the first half reaction (acylation) and full reaction (cyclisation), as using aa-DBE is expected to increase the energy barrier for the reaction it participates in, being the first acylation step or the second half reaction to generate a cyclic peptide product. Clear differences between *Para*CDPS and *Parcu*CDPS were observed when we analysed progress curves monitoring cyclic peptide formation in hybrid reactions with aa-DBE and aa-tRNA (Fig. 3d). *Para*CDPS displayed a small difference in the rate of product formed in the first hour of reaction when using ^Phe^tRNA_Phe_ or Phe-DBE as substrate, suggesting the rate for the first half reaction is similar for both substrates and that cyclisation is rate determining. This points towards a less significant role of aa-tRNA in substrate positioning for the first half reaction. Conversely, the slowest step of the reaction catalysed by *Parcu*CDPS is likely to be in the second half reaction, after substrate is productively bound to P1, and the first acyl-enzyme intermediate is formed. This is because in the reaction catalysed by *Parcu*CDPS having a single DBE substrate in either pocket significantly reduces the yield of cHP.

### Structure of wild type *Parcu*CDPS

**Fig. 4:**
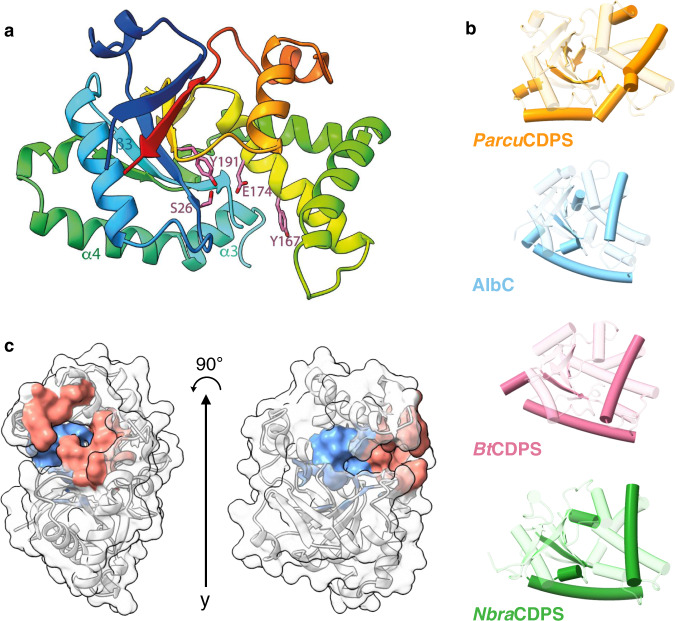
Structure of *Parcu*CDPS. **a** Structure of *Parcu*CDPS coloured rainbow (blue N terminus to red C terminus) and active site residues (pink). **b** Secondary structure comparison of *Parcu*CDPS to three other CDPSs: AlbC, BtCDPS and *Nbra*CDPS. The common CDPS core is shown as transparent colour whilst the major differences are 100% opaque. **c** Pocket volumes of both P1 (blue) and P2 (red) in *Parcu*CDPS are displayed here as calculated by CASTp^38^.

Following these experiments we focused on understanding histidine selection, and more specifically on P1, as it was predicted to possess a narrower binding pocket. To do this, we solved the crystal structure of *Parcu*CDPS and explored the residues determining substrate selection. *Parcu*CDPS belongs to the XYP sub-group of the CDPS family, characterised by the presence of 3 residues: X40 where X is a non-conserved residue, Y202, and P203 (numbering respective to AlbC)^8^. Structures of three previous members from XYP are available^16^, but these enzymes use relatively hydrophobic and non-polar amino acids such as glycine, alanine and leucine. We solved the crystal structure of *Parcu*CDPS at a resolution of 1.90 Å (9 residues out of the total 230 were not traceable), uncovering unique characteristics of histidine substrate selection (Fig. 4a). The structure was solved by iodide SAD phasing after extensive failed trials of molecular replacement, suggesting substantial deviation from previously determined CDPS structures.

**Fig. 5:**
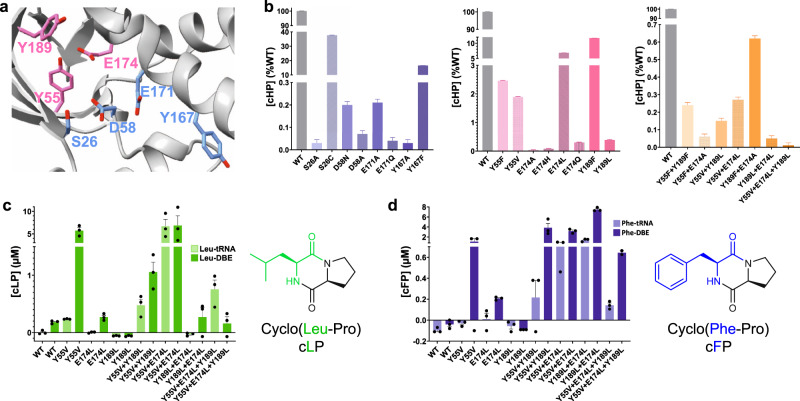
Rational engineering of *Parcu*CDPS P1. **a** enhanced image of the WT structure highlighting the two sets of residues targeted for mutagenesis – active site (blue) and pocket 1 (pink). **b** cHP activity assay for each set of mutants using the tRNA pool and purified aaRSs mentioned previously. The activity is shown as a percentage of the wild-type *Parcu*CDPS activity, and errors were propagated accordingly to account for errors in each parameter individually. Each residue is shown in a different colour and the patterned bar represents a second mutation of the same residue. The overall trend shown is a decrease in the capability of the mutants to produce cHP with S26C displaying the overall highest yield. **c** Quantification of cyclo(Leu-Pro) production using *Parcu*CDPS variants; Pro-tRNA was used in combination with Leu-tRNA and Leu-DBE to investigate the use of different substrates on product yield. **d** Quantification of cyclo(Phe-Pro) using the same mutants as tested for cLP. Data is shown on panels **b**, **c** and **d** as the individual points + standard error of the mean (SEM) of experiments conducted in triplicate, measurements were taken from distinct samples, and authentic commercial standards were used for calibration curves and quantification.

*Parcu*CDPS displays a Rossman-fold common throughout the CDPS family. The active site includes the four conserved residues previously identified: S26, Y167, E171 and Y191 (Fig. 4a)^16^. Additionally, we hypothesised D58 was acting as a potential active site residue, within hydrogen bond distance from the catalytic serine (S26), which could be important for S26 to act as a nucleophile. Indeed, when the hydrogen bond between S26 and D58 is disrupted by mutating D58 to either an alanine or an asparagine, *Parcu*CDPS loses over 90% of activity, while preserving its structure (Fig. 5b). Other examples of this interaction are observed in Phospholipase A2 which possesses a Ser/Asp dyad^37^, and future work could be directed towards better understanding the catalytic mechanism of *Parcu*CDPS. Moreover, the side chain of the predicted catalytically important Y167 points away from the active site, removing the conventional hydrogen bonding network seen in other CDPSs. Y167 plays the same role as Y178 in AlbC which is hypothesised to stabilise the aminoacyl moiety formed from the binding of the first substrate to P1. Further comparison with other CDPSs from the XYP family reveals RMSD values ranging from 2.7 to 2.9 Å (Supplementary Note 3 and Supplementary Fig. 12 for structural imposition). This structural comparison highlighted a divergence in secondary structure and Fig. 4b depicts the core fold in transparent colour and the divergent regions shown in solid colour. *Parcu*CDPS structure diverges from the common fold in helix α3 and beta-strand β3, where these regions change direction. In the common CDPS fold helix α3 and α4 exist as a single continuous helix whereas, in *Parcu*CDPS a flexible glycine residue (G84) provides a significant bend and change of direction, splitting the helix into two. β3 holds another important deviation seen in *Parcu*CDPS’ structure, as it changes direction (compared to common CDPS fold) at I56 to divide the active site pocket. This structural change of β3 facilitates the placement of D58 into H-bonding distance of the active site S26. These two features differentiate *Parcu*CDPS from the previously solved CDPS structures which are fairly conserved with respect to each other.

### Unique characteristics of binding pockets P1 and P2

CASTp^38^ was used to investigate the pocket volume of *Parcu*CDPS and highlighted pocket residues which are unique^9^. P1 (shown in blue in Fig. 4c) is found deeper within the enzyme and is smaller and more restricted in size than P2 which sits at the solvent-accessible edge of the CDPS (red in Fig. 4c). The large size of P2 further explains poor positioning of Pro-DBE as the substrate can potentially sample several conformations, most of which are likely unproductive.

We used PROPKA^39^ to calculate theoretical pKa values for residues in our model at pH 7.0 and generated electrostatic potential maps for each protein variant (calculated data found in Supplementary Note 6 and Supplementary Table 4). From these calculations, P1 is predicted to be mostly neutral apart from Tyr55 and Glu174 which form a negatively charged microenvironment, while P2 is predicted to be mostly lined by positively charged residues. The more positively charged section of P2 could be facilitating the production of cHE—which is also a product of *Parcu*CDPS (Supplementary Note 2). Glutamate is a large flexible amino acid, likely negatively charged at reaction pH^40^. Thus participation in electrostatic interactions of the ^Glu^tRNA_Glu_ substrate with positively charged residues in P2 is plausible, while specific interactions with ^Pro^tRNA_Pro_ are less obvious. Interactions with negatively charged groups in the tRNA are also possible. Although a low resolution complex structure between ^Phe^tRNA_Phe_ and the CDPS from *Candidatus Glomeribacter gigasporarum* indicates no such interactions occur on P2^41^, the structure of *Parcu*CDPS is significantly divergent and a different tRNA binding orientation could occur.

### Rationally altering substrate selection by *Parcu*CDPS

Previous research by us and others has shown that mutations in active site residues seriously reduce product formation in CDPS enzymes. However, altering the substrate scope of a CDPS by mutating select residues has been unsuccessful (recently reviewed in ref. ^10^). Inspired by HisRS and histidine recognition more generally (Supplementary Note 8), and by the only high resolution structure of a CDPS with a ligand bound^12^, we systematically altered three unique residues in *Parcu*CDPS P1, which we hypothesised to be participating in crucial interactions with the polar side chain of histidine. We first designed Generation 1 containing seven P1 mutants (Y55F; Y55V; E174A; E174H; E174L; Y189F and Y189L), and these variants were cloned, expressed in *E.coli*, purified and used for activity assays. The presence of the mutation was confirmed using intact protein mass spectrometry (Supplementary Fig. 2) and the enzymatic activity was probed using LC-MS as previously described. The active site mutants – S26A; S26C; D58A; D58N; Y167A; Y167F; E171A; and E171Q—were also generated to confirm the loss of activity upon removing catalytic residues (Fig. 5a).

All mutants were probed for cHP production using the same activity assay performed on the WT as well as the trapped acyl-enzyme intermediate assay (Supplementary Fig. 6). This assay involved the use of the purified tRNA pool in conjunction with the amino acid tRNA synthetases (HisRS and ProRS) to yield cHP. Figure 5b shows that only S26C and Y167F from the active site variants could still produce cHP albeit with a lower yield. This result is akin to the trend in activity published by Bourgeois et al. who also mutated the catalytic Tyr in three different XYP CDPSs which still produced their respective products^16^. This demonstrates that the phenyl ring is vital in substrate binding rather than the hydrogen bonding interactions from the phenolic hydroxyl^12,15^. When the H-bond between S26 and D58 is disrupted by mutating D58 to either an alanine or an asparagine, the enzyme is inactive (activity < 1% WT). This highlights the essential role that D58 has in potentially polarizing and positioning the serine in the active site for substrate binding and acyl enzyme formation. By mutating the P1 residues, the activity of the variants was vastly reduced with E174A and E174H incapable of tolerating histidine as a substrate. E174A removed the hydrogen bonding network thought to be essential for steering histidine into the pocket whilst E174H was designed to reverse the charge of the residue and was predicted to repel the incoming histidine from P1. Further investigation into the changes imposed by these mutants was performed by solving the crystal structures of a select few mutants containing changes to the active site or the pocket residues. The mutant structures were able to be solved using WT as a model and retained an equivalent fold to *Parcu*CDPS (Supplementary Notes 4 and 7 relating to Supplementary Figs. 13 and 14). Therefore, the disruption of activity is caused exclusively by the change in these few residues which appear important for the enzyme to produce a CDP.

Following on from these results, we designed Generation 2 of double mutants from P1 residues (variants Y55F **+** Y189F, Y55F **+** E174A, Y189F **+** E174A, E174L **+** Y189L), aiming to severely perturb the activity of the enzyme towards histidine. When we investigated the formation of cHP using Generation 2 variants, all mutants synthesised significantly less cHP than the single Gen 1 mutants alone (Fig. 5b). This suggests that binding to the P1 pocket is not facilitated by a single residue alone, rather by a combination of interactions. The impact of mutating Y55 carries more weight than Y189, suggesting it may directly interact with the histidine residue via a H-bond rather than adding to the polar surface. A similar pattern of residues interacting with the imidazole from histidine is seen in HisRS, in which two tyrosine and a glutamate residue mediate interactions with the nitrogen groups of histidine (Supplementary Fig. 15).

**Fig. 6:**
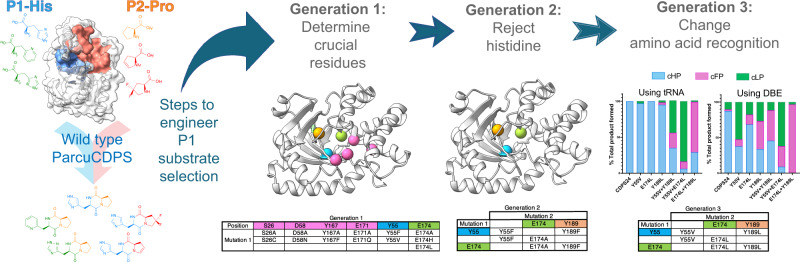
Swapping the substrate selection on P1 by *Parcu*CDPS. Based on P1 residues we produced a series of rationally designed mutants to determine residues crucial for histidine recognition (Generation 1); reject histidine as a substrate (Generation 2) and select a different amino acid on P1 to produce cyclic dipeptides that no longer contain histidine (Generation 3).

Having hindered the capacity of *Parcu*CDPS to recognize histidine, we then focussed on Generation 3 composed of three double mutants (Y55V **+** E174L; Y55V **+** Y189L; E174L **+** Y189L) and a triple mutant (Y55V **+** E174L **+** Y189L), aimed at switching the substrate specificity to a less polar amino acid. This was because Generation 1 and 2 variants essentially incorporate hydrophobic and non-polar amino acids potentially altering the overall environment and electrostatics of P1. Therefore, we hypothesised that whilst these enzymes were incapable of cHP production, they could tolerate a different, less charged substrate in P1. Previously we showed that CDPSs can use both tRNA and DBE substrates to yield a CDP and so using a combination of these, the production of cLP and cFP by *Parcu*CDPS mutants was investigated (Fig. 5c, d). Interestingly, reactions using ^Pro^tRNA_Pro_ and Leu-DBE/Phe-DBE gave a higher yield of product compared to ^Pro^tRNA_Pro_ and ^Leu^tRNA_Leu_/^Phe^tRNA_Phe_. This indicates that the enzyme is still able to recognise and reject the tRNA body, which can be circumvented by using a smaller DBE substrate. It is evident that whilst wild-type *Parcu*CDPS is unable to use either leucine or phenylalanine as substrates, the new mutants can accept these amino acids. All double mutants were capable of producing cLP and cFP albeit with varying yields: 84% of the total products formed by Y55V **+** E174L was cLP, whilst for E174L **+** Y189L, 70% of products formed was cFP. These mutants, however, did not accept other small hydrophobic amino acids such as valine and isoleucine thus demonstrating that the enzyme is still actively selecting its substrates. Trapped acyl enzyme intermediate experiments confirmed this trend of switched amino acid selection (Supplementary Fig. 6). Overall, this is the first report of a CDPS displaying a change in substrate specificity using targeted enzyme engineering. Figure 6 summarizes the steps required to achieve this shift. The products of the *Parcu*CDPS variants with changed specificity—cLP and cFP—are biologically relevant molecules with known applications as anti-cancer drugs. Jinendiran *et al*. reported cell death of colorectal cancer cells (HT-29) in zebrafish xenograft model after dosing with either CDP^42^. This finding showcases the advantages of mutating a CDPS to produce interesting molecules with untapped potential.

## Conclusions

We set out to investigate the cyclodipeptide-synthesising capability of two cyclodipeptide synthases which accept histidine as a substrate. Our work uncovered that the use of a collective tRNA pool was sufficient for the CDPSs to yield their expected product in addition to accepting a variety of unnatural amino acids as substrates for CDP formation. This in vitro method has many advantages such as reaction scalability and has proven useful for generating cyclic dipeptides containing both canonical and non-canonical amino acids.

Additionally, structural characterisation of *Parcu*CDPS revealed a so far unique pocket topology to accommodate histidine as a substrate. By trapping the acyl-enzyme intermediate we determined that histidine was bound in P1 of *Parcu*CDPS but on P2 for *Para*CDPS, stressing differences between the two enzymes, and more broadly on the rate limiting nature of different steps in the reactions they catalyse.

Finally, combining structural biology and activity assays, we provide a much clearer picture of how polar residues such as histidine are selected by cyclodipeptide synthases as substrates on P1, as well as how this selectivity can be manipulated by rational engineering to produce molecules of our choosing. pKa calculations using experimentally determined structures reveal that several residues in proximity are likely altering electrostatics of the binding pocket and therefore influencing substrate selection. Although product yield by a CDPS protein has been improved by engineering P1^43^, there are no published attempts to alter the substrate scope of a CDPS enzyme. Therefore, this is a pivotal finding which could lead to a wide array of CDPs from a single engineered enzyme, taking control and manipulating substrate selection by these enzymes.

## Methods

### General

All reagents used here were purchased at the highest commercial quality (>95%) and used without further purification unless otherwise specified. Protein purifications were performed on HisTrap HP columns (GE Healthcare) followed by size exclusion chromatography on a Superdex 200 Increase 16/60 in the relevant buffers. In vitro enzymatic assays were performed as described in the Supplementary Methods and analysed using a Waters ACQUITY UPLC liquid chromatography system equipped with an electrospray ionisation (ESI) source. All X-ray diffraction data was obtained at Diamond Light Source in Oxford, UK and the data was processed and refined using PHENIX. Amino acid-DBE substrates were synthesised following a published method and ^1^H NMR spectra was recorded on a Bruker AV 400 equipped with a BBFO probe. Full experimental details can be found in the Supplementary Methods section of the Supplementary Information.

### Reporting summary

Further information on research design is available in the Nature Research Reporting Summary linked to this article.

## Supplementary information

Czekster_PR File

Supplementary information

Description of Additional Supplementary Files

Supplementary Data 1

Supplementary Data 2

Supplementary Data 3

Supplementary Data 4

Supplementary Data 5

Supplementary Data 6

Supplementary Data 7

Reporting Summary

## Data Availability

All data supporting the findings of this research are available within the article and its corresponding supplementary information (SI) file. Full details of experimental methods can be found in the SI under the heading ‘Supplementary Methods’. For mass spectrometry of the unnatural amino acids tested here, see Supplementary Note 1. For HPLC analysis of *Parcu*CDPS product formation, see Supplementary Note 2. For crystallographic information of *Parcu*CDPS, see Supplementary Notes 3-5. The crystallographic data table can be found in Supplementary Note 5 (Supplementary Table 3) and the Protein Data Bank (PDB) accession codes of each protein structure detailed herein are as follows: 7QB8 (*Parcu*CDPS-WT, Supplementary Data 1); 7QAY (*Parcu*CDPS-Y55F, Supplementary Data 2); 7QAU (*Parcu*CDPS-D58N, Supplementary Data 3); 7QAX (*Parcu*CDPS-E171Q, Supplementary Data 4); 7QAQ (*Parcu*CDPS-E174A, Supplementary Data 5); 7QAT (*Parcu*CDPS-E174L, Supplementary Data 6); 7QAW (*Parcu*CDPS-Y189F, Supplementary Data 7). For additional information complementary to this article, see Supplementary Notes 6-9. Full datasets from LC-MS are available online at Figshare under the Project: Active site remodelling of a cyclodipeptide synthase redefines substrate scope. Alternatively, individual datasets can be found as indicated below: Fig. 2a - 10.6084/m9.figshare.c.6131892.v1; Fig. 2c - 10.6084/m9.figshare.c.6131892.v1; Fig. 3b - ; Fig. 3d - 10.6084/m9.figshare.c.6131892.v1; Fig. 5a - 10.6084/m9.figshare.c.6131892.v1; Fig. 5c - 10.6084/m9.figshare.c.6131892.v1; Fig. 5d - 10.6084/m9.figshare.c.6131892.v1.
