# Supplementary material for: Active site remodelling of a cyclodipeptide synthase redefines substrate scope
Source: Commun Chem. 2022 Aug 25;5:101. doi: 10.1038/s42004-022-00715-2 (PMC7613923; doi:10.1038/s42004-022-00715-2)
Supplement: Supplementary file 2 — Supplementary information [file 42004_2022_715_MOESM2_ESM.docx]

Active site remodelling of a cyclodipeptide synthase redefines substrate scope

E. Sutherland, C. J. Harding and C. M. Czekster

**Supplementary Information**

[Supplementary Methods 2](#_Toc108798365)

[Cloning, expression, and purification of CDPS enzymes 2](#_Toc108798366)

[Crystallography of *Parcu*CDPS 5](#_Toc108798367)

[tRNA Pool Extraction 6](#_Toc108798368)

[Purification of amino acid tRNA synthetases (aaRS) 6](#_Toc108798369)

[S30 Extract Preparation 7](#_Toc108798370)

[Cyclodipeptide Synthase Activity Assay 8](#_Toc108798371)

[Activity Assay using DBE Substrates 9](#_Toc108798372)

[Cyclic Dipeptide Identification via LC-MS 10](#_Toc108798373)

[Synthesis of Amino-DBE Compounds – His-DBE and Phe-DBE 10](#_Toc108798374)

[Trapped acyl-enzyme intermediate 12](#_Toc108798375)

[Supplementary Note 1 - Mass Spectrometry of Unnatural CDP Library 13](#_Toc108798376)

[Supplementary Note 2 - HPLC Analysis of cHE and cHP 22](#_Toc108798377)

[Supplementary Note 3 - Superimposition of *Parcu*CDPS with most similar known CDPS……………………………………………………………………………………………………………………………………..………23](#_Toc108798378)

[Supplementary Note 4 - *Parcu*CDPS Mutant Crystal Structures 23](#_Toc108798379)

[Supplementary Note 5 - *Parcu*CDPS Crystallographic Data Table 24](#_Toc108798380)

[Supplementary Note 6 - Comparison of mutant pKa values 26](#_Toc108798381)

[Supplementary Note 7 - Differential scanning fluorimetry of *Parcu*CDPS mutants 27](#_Toc108798382)

[Supplementary Note 8 - Histidine binding pocket comparison 28](#_Toc108798383)

[Supplementary Note 9 - Novel cyclodipeptides 29](#_Toc108798384)

[Supplementary References 30](#_Toc108798385)

# **Supplementary Methods**

## Cloning, expression, and purification of CDPS enzymes

The CDPS genes – *Para*CDPS from *Parabacteroides sp. 20_3* and *Parcu*CDPS from *Parcubacteria bacterium RAAC4_OD1_1* – were synthesised as a codon optimized for *E. coli* expression gBlock from IDT. The synthesised genes were inserted into a pJ411 expression plasmid with a C-terminal 6xHis protein tag by Gibson assembly cloning technique.[2](#_ENREF_2) Mutants of *Parcu*CDPS were created by site-directed mutagenesis based on the NEB Q5 site-directed mutagenesis kit. Presence of both the WT and mutant were confirmed via sequencing before cloning into the commercially available *E. coli* BL21(DE3) expression strain (NEB). Cells were grown at 37 °C until the OD_600_ had reached 0.6 and protein expression was induced using IPTG (1mM). The cells were then grown at 16 °C overnight.

After harvesting, the resultant protein pellet was purified, first by resuspending in the appropriate volume of lysis buffer (50 mM HEPES pH 7.0, 250 mM NaCl, 20 mM imidazole, 5% glycerol). The solution was lysed using a high-pressure cell disruptor (Constant Systems) before being centrifuged at 51000g for 30 minutes, 4 °C. The cell lysate was filtered through an 0.8 µm membrane and loaded onto a 5 mL HisTrap HP column (GE Healthcare), pre-equilibrated with lysis buffer. The column was washed with 20 column volumes (CV) of lysis buffer and the adsorbed proteins were eluted using elution buffer (50 mM HEPES pH 7.0, 250 mM NaCl, 300 mM imidazole, 5% glycerol) in 3 steps: 10%, 20% and 100%. Proteins of interest were dialysed into dialysis buffer (20 mM HEPES pH 7, 250 mM NaCl, 5 mM 2-mercapoethanol) overnight at 4 °C. The wild-type proteins were purified further using size exclusion chromatography in the dialysis buffer mentioned previously. (Superdex 200 Increase 16/60). Enzyme activity was determined following assay conditions as described on page 7-8 and cyclic product was detected using liquid chromatography mass spectrometry (LC-MS).

**a**


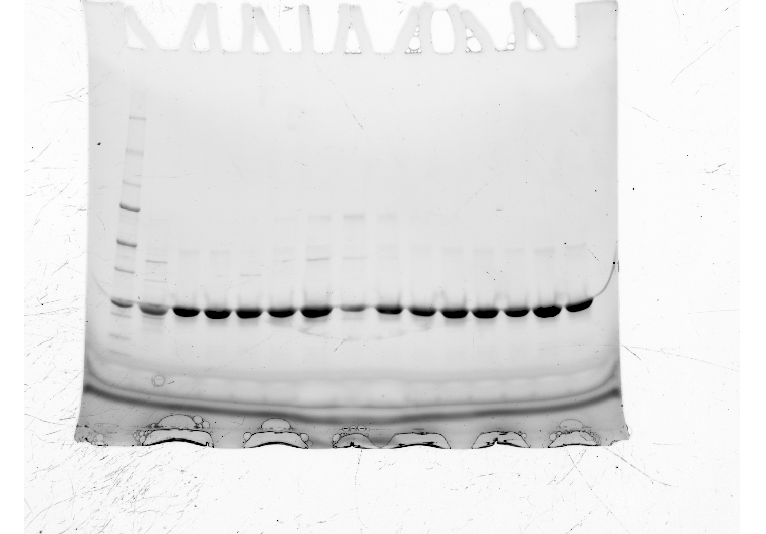


1 2 3 4 5 6 7 8 9 10 11 12 13 14


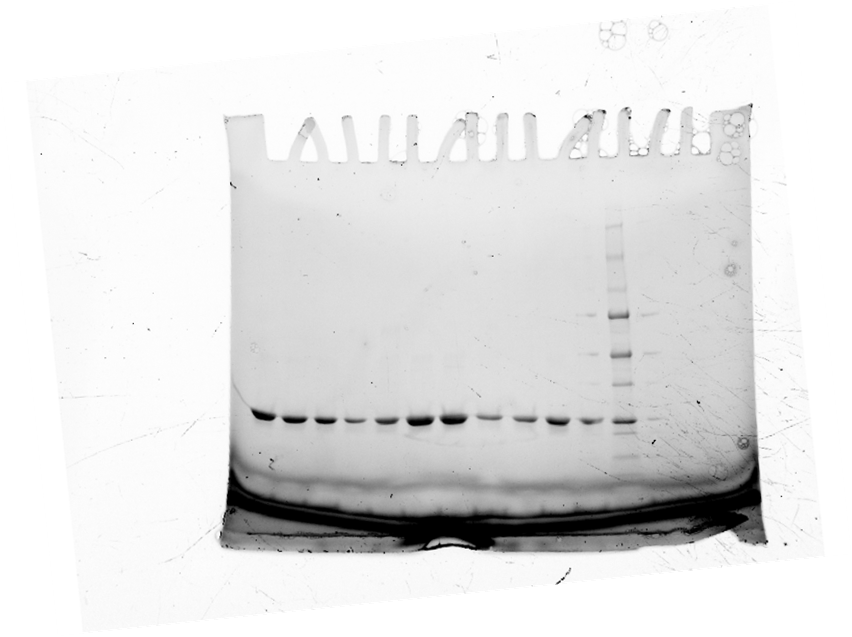


15 16 17 18 19 20 21 22 23 24

**
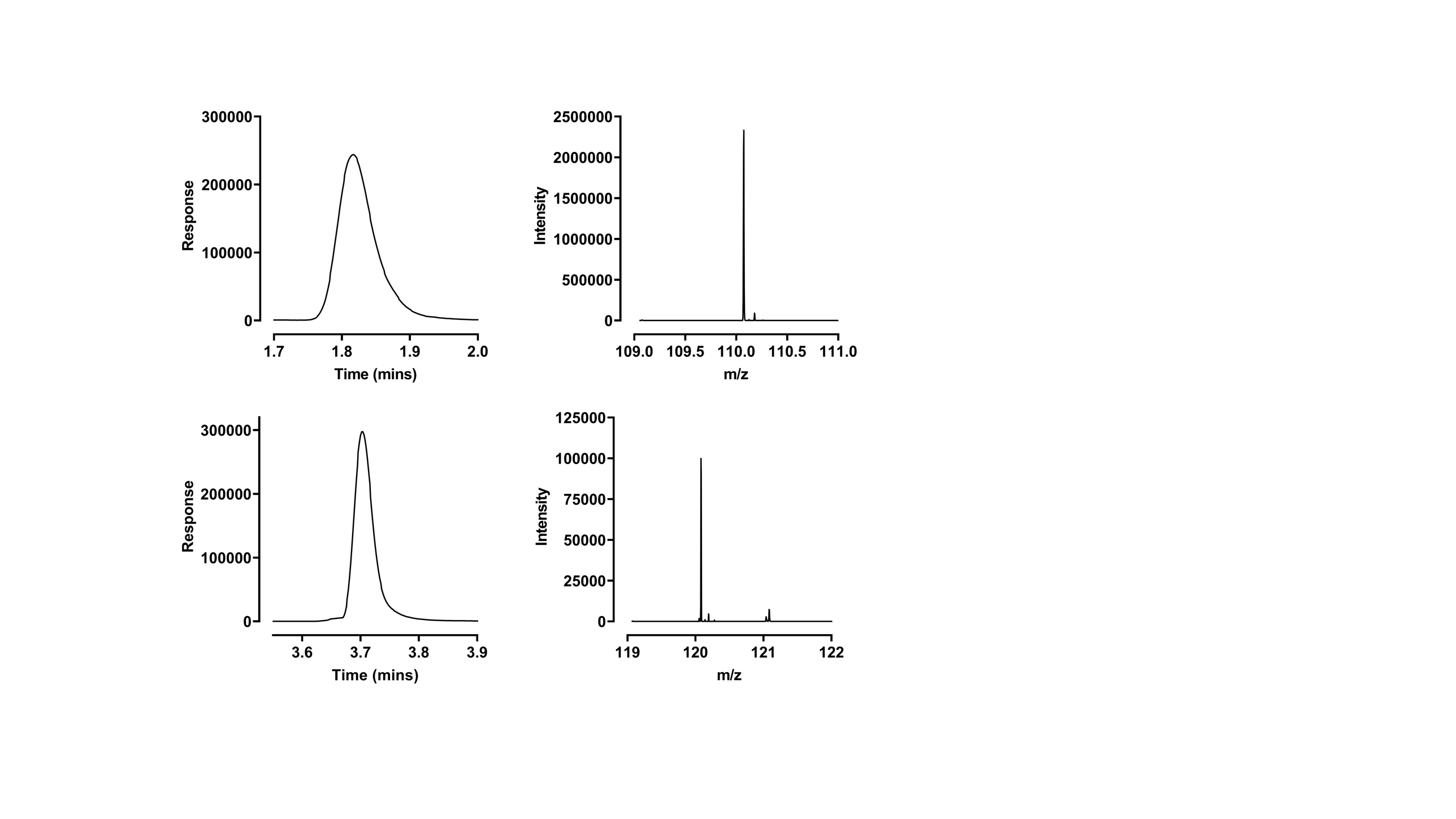

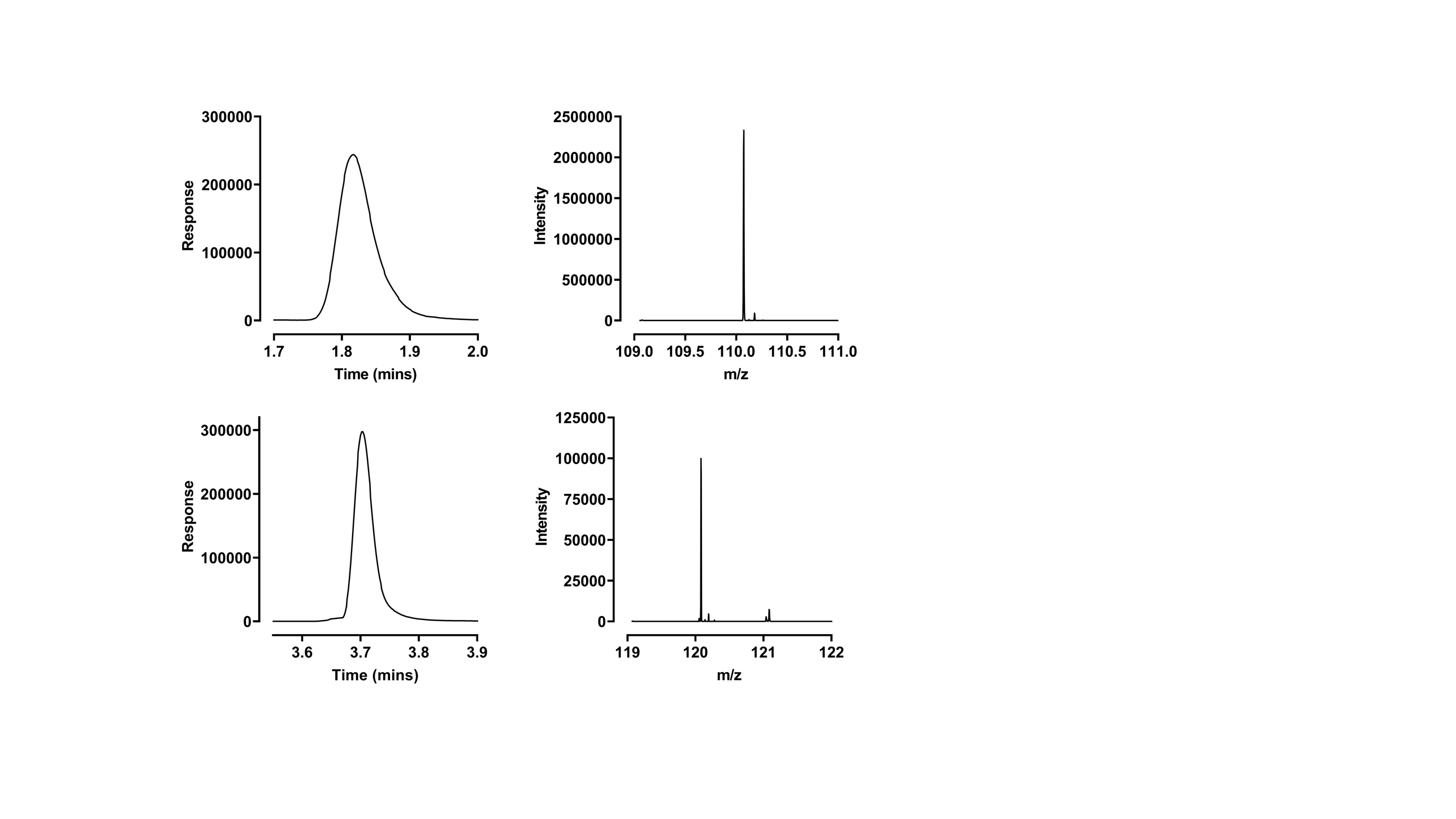
**

**b**

**c**

**Supplementary Fig. 1a** Purification of all CDPS proteins studied here. Proteins are shown after final purification step via SDS-PAGE analysis loaded at 20 µM. The identity of the lanes are as follows: 1 – *Para*CDPS; 2 – *Parcu*CDPS; 3 – S26A; 4 – S26C; 5 – Y55F; 6 – Y55V; 7 – D58A; 8 – D58N; 9 – E171A; 10 – E171Q; 11 – E174A; 12 - E174H; 13 – E174L; 14 – Y167A; 15 – Y167F; 16 – Y189F; 17 – Y189L; 18 – Y55F + Y189F; 19 – Y55F + E174A; 20 – Y55V + Y189L; 21 - Y55V + E174L; 22 -E174A+ Y189F; 23 - E174L + Y189L; 24 - Y55V + E174L + Y189L. Any variants shown here are from mutagenesis of *Parcu*CDPS. **1b** LC-MS analysis of activity assay using purified *Parcu*CDPS and *in vitro* transcribed His-tRNA and Pro-tRNA with purified HisRS and ProRs. Product was detected using an MRM method detecting a unique transition of cHP (m/z 235.1190 to 110.0741). **1c** LC-MS analysis of activity assay using purified *Para*CDPS and *in vitro* transcribed His-tRNA and Phe-tRNA with purified HisRS and PheRS. Product was detected using an MRM method detecting a unique transition of cHF (m/z 285.1246 to 120.0814).

**Amino acid sequence of *Para*CDPS:**

MSLLYERRLESCYIERIYPYSESNAFLGVSICSRLFSEKTLIALFDWCKVNVKSVYVLIADEIQMYTFMASKGLERKEACAKALQIGDIKYRFIERVIKKGDYDNVRLLSWKAVALEPRFKTLLQRLRLLYGTEILFRREVIKQILERNRRLPEGFRFARIDSSDYDLASLYILNELAVILYFHLYFDPVCQYQISPLPMTPLLEILYDGTFLKDLLVPKKDIGYIEIMEEKDLTGRMIKVNTKGAPLEHHHHHH

**Amino acid sequence of *Parcu*CDPS:**

MELHQIRGCHKNDIELKKYNIGVAISLGNKWFSIDNIEKLVKWSLLHTKEYVIIYIADSIHGINLSVRNKLSDSHAEEVAIRYGRNLFIKIKERVSLSFSQDEQAKIIYATWSDIADSKYKEKVKYLYNLYDKNINFKNYIENFVKEWVSKEKRTFNNNEINKFGRYILEELPELMVQVKARGVLFEAYVYPYKTRITEFVGLLQKGEIFPEIKTNILDNHPKIFLEVREHHHHHH

(C-terminal 6xHis tag shown in red)

**
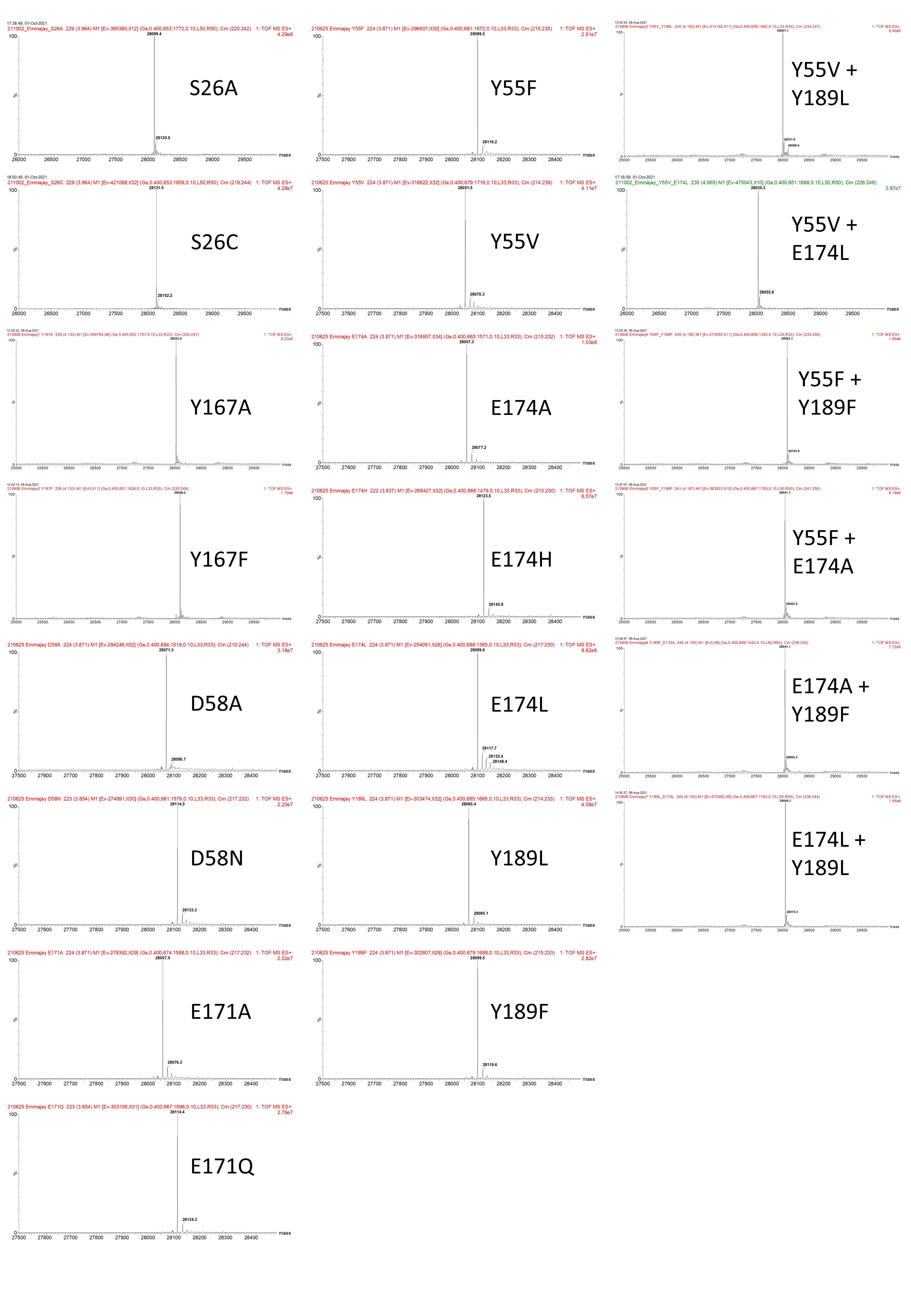
Intact Mass Spectrometry of *Parcu*CDPS Mutants**

**Supplementary Fig. 2** Intact protein mass spectrometry analysis of *Parcu*CDPS variants used to confirm the desired mutation was present.

## Crystallography of *Parcu*CDPS

Crystals of *Parcu*CDPS and mutants were grown at 4 °C using the sitting drop vapour diffusion technique. Equal volumes of protein and reservoir solution (0.15 µL) were used. Initial attempts to crystallise *Parcu*CDPS in its original storage buffer were unsuccessful which led to a repeat purification to change the storage buffer to 50mM citrate pH 6.5, 150mM NaCl, 2mM DTT and 2mM EDTA. From this new prep, crystals of *Parcu*CDPS were grown in 2.27 M ammonium sulphate and 0.1 M sodium acetate pH 5.13. Crystals were cryoprotected in mother liquor supplemented with 10% (v/v) ethylene glycol and stored in liquid nitrogen. Initial phasing of wild-type *Parcu*CDPS was achieved by soaking crystals in mother liquor supplemented with 0.5M NaI for 5 minutes before being fished – the structure was consequently solved using an iodine-SAD data set. The subsequent mutants of *Parcu*CDPS were solved using the wild-type as the molecular replacement model in PHASER.[3](#_ENREF_3) X-Ray Diffraction data was collected at the Diamond Light Source in Oxford, UK. Protein structures were built and refined using COOT, optimised using PDB-redo and this refinement was assessed in PHENIX.[3-5](#_ENREF_3)

**tRNA Preparation**

tRNA of interest was synthesised following the *in vitro* transcription protocol described by Beckert *at al.*[6](#_ENREF_6) The template DNA of tRNA from *E. coli* and the respective organisms of the CDPSs (*Parabacteroides* and *Parcubacteria bacterium RAAC4_OD1_1*) was amplified by PCR. *In vitro* transcription composed of 20 mg PCR product, 20 mM MgCl2, 50 mM HEPES pH 7.5, 2 mM Spermidine, 20 mM DTT, 5 mM ATP, 5 mM UTP, 5 mM CTP, 6 mM GTP, 5 mM RNA polymerase Delta172–173_49_ was incubated at 37 °C overnight. Following this, DnaseI (Rnase-free, NEB) was added and incubated for 1 hour at 37 °C. To confirm the presence of tRNA, a urea-TBE-PAGE gel was prepared using the standard protocol and the samples were analysed before further purification. A phenol:chloroform (5:1) extraction and a 70% EtOH precipitation was appropriate to remove any contaminants before resuspending the tRNA pellet in DEPC treated water. The concentration of the resultant tRNA was calculated using their A260 values and the Beer-Lambert law.


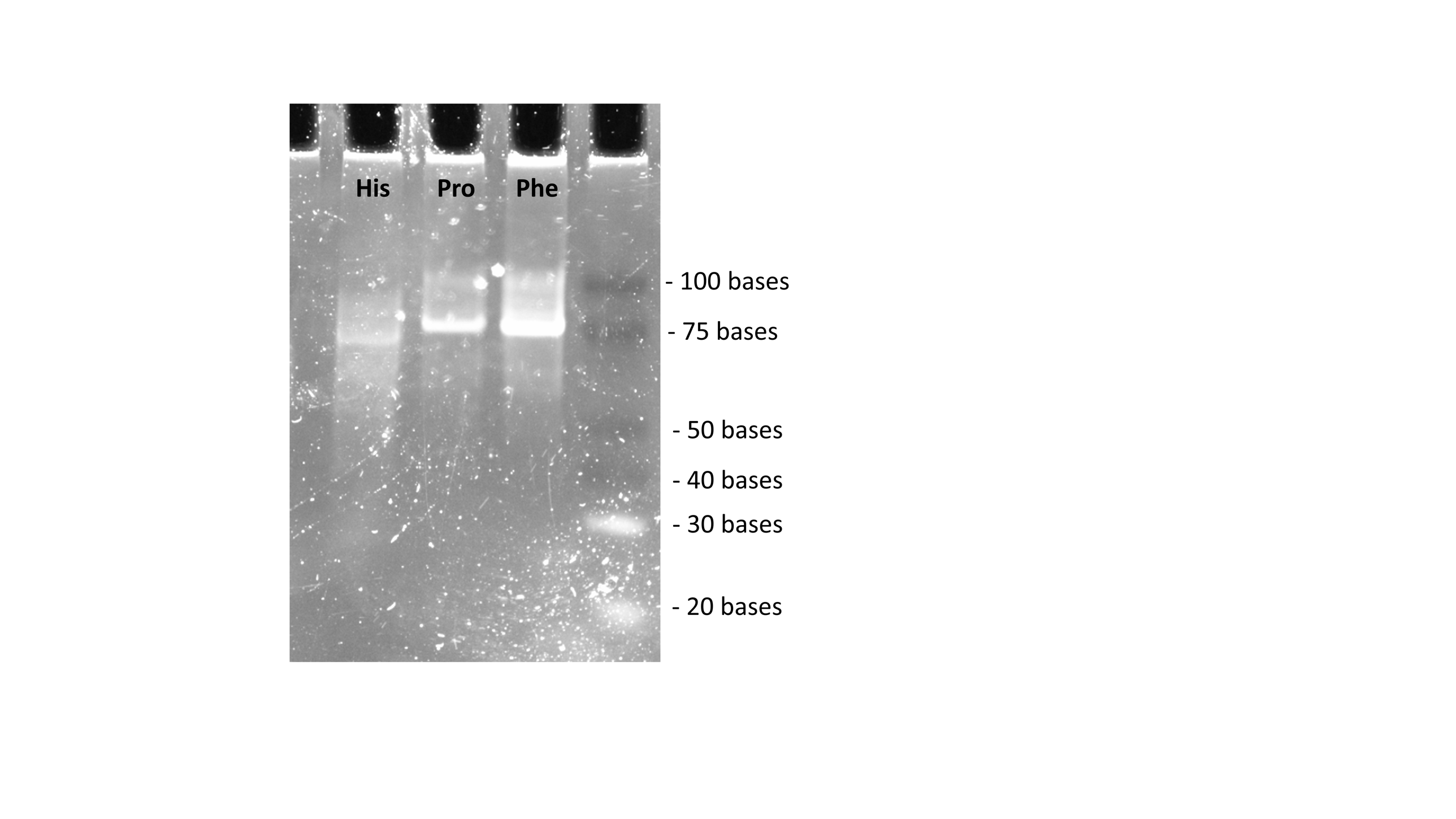


**Supplementary Fig. 3** RNA denaturing gel ran in 1X TBE then stained with SYBRSafe dye. The ladder used was DynaMarker® Prestain Marker for Small RNA Plus annotated as shown. The expected length of the RNA are as listed: 75 bases – His; 77 bases – Pro; 76 – Phe.

| **tRNA** | **RNA Sequence** |
| --- | --- |
| His | GUGGCUAUAGCUCAGUUGGUAGAGCCCUGGAUUGUGAUUCCAGUUGUCGUGGGUUCGAAUCCCAUUAGCCACCCCA |
| Pro | CGGUGAUUGGCGCAGCCUGGUAGCGCACUUCGUUCGGGACGAAGGGGUCGGAGGUUCGAAUCCUCUAUCACCGACCA |
| Phe | GCCCGGAUAGCUCAGUCGGUAGAGCAGGGGAUUGAAAAUCCCCGUGUCCUUGGUUCGAUUCCGAGUCCGGGCACCA |

## tRNA Pool Extraction

To extract the pool of all tRNA produced by *E. coli*, a method was adapted from a protocol described by Mechulam *et al.*[7](#_ENREF_7) Firstly, an overnight culture was prepared from commercially available BL21 DE3 cells in lysogeny broth (LB). Terrific broth (TB, 1L) was inoculated from this and split into two separate flasks before being incubated at 37 °C until the OD_600_ had reached 4. The cells were pelleted via centrifugation (6000 RPM, 15 minutes, 4 °C) and resuspended in a buffer containing 1 mM HEPES-KOH, pH 7.5 and 10 mM magnesium acetate (8.6 mL). Phenol:chloroform (5:1) was added to the suspension and the mixture was centrifuged (15000 RCF, 30 minutes, 20°C). The top layer containing RNA was moved to a new falcon tube where 0.1 volumes of 5M NaCl and 2.2 volumes of cold ethanol were added. The mixture was centrifuged (15000 RCF, 30 minutes, 4 °C) and the supernatant was discarded. The pellet was resuspended in 1 M NaCl (5 mL) and the solution was centrifuged (15000 RCF, 30 minutes, 4 °C). The supernatant was transferred to a new falcon tube and the tRNA was precipitated using 2 volumes of cold ethanol. The solution was centrifuged (15000 RCF, 30 minutes, 4 °C) and the pellet was dissolved in 100 mM HEPES-KOH, pH 8 (2 mL). Deacylation of tRNA was performed by incubating at 37 °C for one hour followed by precipitation using 0.1 volume of 5 M NaCl and 2.2 volumes of cold ethanol. The solution was centrifuged (4000 RPM, 10 minutes, 4 °C) and the pellet was resuspended in 70% cold ethanol (2 mL) to perform an overnight precipitation at -80 °C. Following incubation, the tRNA was pelleted via centrifugation (4000 RPM, 10 minutes, 4 °C) and resuspended in DEPC-treated water (4 mL). The concentration of this tRNA pool was calculated using the Beer-Lambert where the extinction coefficient is a sum of the extinction coefficients for the four RNA nucleobases present.

## Purification of amino acid tRNA synthetases (aaRS)

1. HisRS

pET21a-HisRS-His was a gift from Sebastian Maerkl & Takuya Ueda (Addgene plasmid # 124111 ; http://n2t.net/addgene:124111 ; RRID:Addgene_124111)[_ENREF_8](#_ENREF_8).

1. PheRS-A294G
   pKPY514 was a gift from David Tirrell (Addgene plasmid # 62598 http://n2t.net/addgene:62598 ; RRID:Addgene_62598).
2. ProRS was cloned into pJ414 using *E. coli* genomic DNA (gift from Dr Huanting Liu) by Gibson Assembly.
3. GluRS pET21a-GluRS-His was a gift from Sebastian Maerkl & Takuya Ueda (Addgene plasmid # 124109 ; http://n2t.net/addgene:124109 ; RRID:Addgene_124109)
4. LeuRS

Plasmid pET15-LeuRS was a kind gift from Dr Andres Palencia National Institute of Health and Medical Research (Inserm)[8](#_ENREF_8)

aaRS other than HisRS were purified using the same protocol, described below:

**General aaRS purification protocol**

Cell pellets (usually 2-3L grown in LB media) were resuspended (30 mL/1L cultured cells) in buffer A (50mM HEPES pH 8.0, 500mM NaCl, 20mM Imidazole pH8.0). Cells were disrupted using a homogenizer (Constant systems) set at 30kpsi (2 passages). Cell debris were removed by centrifugation at 32000g for 30minutes, supernatant was removed and filtered through a 0.8 μm filter. Filtered lysate was applied onto a 5ml HisTrap column (Biorad) pre-equilibrated with buffer A. After loading, column was washed with 20 CV of buffer A. Protein was eluted with buffer B (buffer A with 300mM Imidazole), and fractions containing the protein of interest (verified by SDS-PAGE) were pooled and Dialysed into 2L buffer (20mM Tris pH7.5, 50mM NaCl, 10mM MgCl_2,_ 7mM β-mercaptoethanol, 10% Glycerol) O/N in cold room. Following dialysis protein was concentrated to 100 or 200 μM, split into 50 μL aliquots and snap frozen using liquid nitrogen.

**HisRS purification protocol**

The purification buffers were adapted from Shimizu *et al.*[9](#_ENREF_9) Expression of HisRS was induced in *E.coli* using IPTG once OD_600_ had reached 0.8. The cells were grown at 37 °C post induction for 4 hours before being harvested. The buffers used for purification ere as follows: Lysis buffer – 50 mM HEPES-KOH pH 7.6, 10 mM MgCl_2_, 10 mM imidazole and 2 mM 2-mercaptoethanol; Elution buffer: 50 mM HEPES-KOH pH 7.6, 10 mM MgCl_2_, 400 mM imidazole and 2 mM 2-mercaptoethanol; Dialysis buffer - 50mM HEPES-KOH pH 7.6, 10 mM MgCl_2_, 100 mM KCl, 7 mM 2-mercaptoethanol and 30% glycerol. Protease tablets (cOmplete Tablets, EDTA free, Roche) were added to the lysis buffer before resuspending the pellet in 180mL. Lysozyme (10mg) and DNase (1mg) were added and the solution was stirred at 4 °C for 30 minutes. The cells were lysed, centrifuged and eluted from a 5mL Nickel column using an imidazole gradient. The protein fractions of interest were pooled together and dialysed overnight at 4 °C. Following on from this, the protein was aliquoted and snap frozen for storage at -80 °C.

## S30 Extract Preparation

The synthesis of a cell-free bacterial lysate was first published by Krinsky *et al.* and this method follows theirs closely.[10](#_ENREF_10) TB (1 L) was inoculated with an overnight culture prepared from BL21-DE3 cells grown in LB at a 1:50 (cells:media) ratio. The cells were grown at 37 °C until OD_600_ reached 4 whereupon the cells were pelleted via centrifugation (7000 RCF, 10 minutes, 4 °C). The pellet was resuspended in 1 litre of S30 buffer containing 10 mM tris acetate pH 7.4, 14 mM magnesium acetate, 60 mM potassium acetate, 1 mM DTT and 0.5 ml/L 2-mercapoethanol. The solution was centrifuged again (7000 RCF, 10 minutes, 4 °C) and the pellet was resuspended in S30 buffer (15 mL). The cells were lysed using a high-pressure cell disruptor at 15 kpsi and 0.1 M DTT was added to the lysate (100 µL for every 10 mL of suspension). The lysate was then centrifuged (24700 g, 30 minutes and 4 °C) to yield the S30 extract in the supernatant. The concentration of this was measured using a spectrophotometer.

## Cyclodipeptide Synthase Activity Assay

To investigate the activity of the CDPS enzymes *in vitro*, the following reactions were performed as detailed. All assays requiring aminoacylation of tRNA_Phe_ used the mutant PheRS-A294G. When *Parcu*CDPS was used, amino acid analogues of histidine and proline were in employed, in combination with ProRS and HisRS. When *Para*CDPS was used, amino acid analogues of histidine and phenylalanine were in employed, in combination with PheRS-A294G and HisRS. Only one pair of amino acid substrates was used at a time, so that only one substrate could be occupying P1 or P2.

The assay contained a buffer composed of 100mM HEPES pH 7.0, 100 mM KCl, 10mM MgCl_2_, 5mM ATP, and 10mM DTT. Respective amino acids were added to a final concentration of 500 µM followed by 50 µM of the tRNA pool purified as described above. Before the enzymes were added to the mixture, the pH of the solution was checked to ensure it was 7. Following any necessary pH correction, DEPC-treated water was added to achieve the desired final volume followed by the addition of tRNA synthetases and CDPS enzyme – both at 5 µM final concentration. The reaction was left to proceed overnight at room temperature.

General description of assay conditions using tRNA pool:

| **Component** | **Final Concentration** |
| --- | --- |
| HEPES pH 7.0 | 100 mM |
| KCl | 100 mM |
| MgCl_2_ | 10 mM |
| ATP | 5 mM |
| DTT | 10 mM |
| Amino acid 1 | 500 µM |
| Amino acid 2 | 500 µM |
| tRNA pool | 50 µM |
| aa-tRNA synthetase 1 | 5 µM |
| aa-tRNA synthetase 2 | 5 µM |
| CDPS | 5 µM |

Using the S30 extract required a different buffer system composed of 55mM HEPES-KOH pH 8.0, 14 mM MgOAc, 50mM KOAc, 155mM NH_4_OAc supplemented with 5mM ATP and the necessary amino acids at 2.5 mM final. 22mg/mL of purified S30 extract was used in conjunction with 5 µM CDPS to yield cyclodipeptide products.

General description of assay conditions using S30 extract:

| **Component** | **Final Concentration** |
| --- | --- |
| HEPES-KOH pH 8.0 | 55 mM |
| Magnesium acetate | 14 mM |
| Potassium acetate | 50 mM |
| Ammonium acetate | 155 mM |
| ATP | 5 mM |
| Amino acid 1 | 2.5 mM |
| Amino acid 2 | 2.5 mM |
| S30 extract | 22 mg/mL |
| CDPS | 5 µM |

To quench the assays, cold methanol was added so the total final volume contained 80% methanol. The samples were incubated at –80 °C for 15 minutes and centrifuged for 10 minutes afterwards. The supernatant from this was moved to a new eppendorf and dried under nitrogen. The resultant residue was reconstituted in LC-MS grade water for analysis.

To monitor the production of the cyclic dipeptide formed by the enzyme, a discontinuous time course of the activity assay was performed in triplicate. The reaction was initiated by adding enzyme and 10 time points were taken (20 µL x 10) ranging from 0 minutes to 300 minutes. For each sample, the methanol extraction protocol was followed and analysed via LC-MS.

## Activity Assay using DBE Substrates

For reactions using two DBE substrates, a simplified version of the previously described CDPS activity assay was employed. The reaction solution included 20 mM HEPES, 500 mM NaCl, 5 mM DTT and the DBE substrates both at 500 µM. For experiments using one DBE substrate with one aminoacylated tRNA substrate, the original activity assay procedure was followed with the substitution of one amino acid and its corresponding synthetase with the aa-DBE substrate. The assay was quenched using the same protein precipitation mentioned above.

| **Component** | **Final Concentration** | **Component** | **Final Concentration** |
| --- | --- | --- | --- |
| HEPES pH 7.0 | 20 mM | HEPES pH 7.0 | 100 mM |
| NaCl | 500 mM | KCl | 100 mM |
| DTT | 10 mM | MgCl_2_ | 10 mM |
| Amino acid 1-DBE | 500 µM | ATP | 5 mM |
| Amino acid 2-DBE | 500 µM | DTT | 10 mM |
| CDPS | 5 µM | Amino acid 1 | 500 µM |
|  |  | Amino acid 2-DBE | 500 µM |
|  |  | tRNA pool | 50 µM |
|  |  | aa-tRNA synthetase 1 | 5 µM |
|  |  | CDPS | 5 µM |

## Cyclic Dipeptide Identification via LC-MS

The activity assays were analysed using a Waters ACQUITY UPLC liquid chromatography system coupled to a Xevo G2-XS QTof mass spectrometer equipped with an electrospray ionization (ESI) source. The samples (10 µL) were loaded onto an HSS-T3 column (2.1 x 100 mm, 1.8 μm, Waters Acquity) and ran at 40 °C. The analytes were separated using a gradient mobile phase from 1%B to 50%B where the two mobile phases consisted of A - 0.1% formic acid in water and B – 0.1% formic acid (F.A) in acetonitrile at a flow rate of 400 μL min^-1^.The capillary voltage was set at 2.5 kV in positive ion mode. The source and desolvation gas temperatures of the mass spectrometer were set at 120 °C and 500 °C, respectively. The cone gas flow was set to 50 L/hr whilst the desolvation gas flow was set at 1000 L/hr. An MS^E^ scan was performed between 50 – 700 m/z where function 1 employed MS analysis whilst function 2 applied a collision energy ramp from 15 to 30 V to perform MS/MS fragmentation. In addition, a lockspray signal was measured and a mass correction was applied by collecting every 10s, averaging 3 scans of 1s each using Leucine Enkephalin as a standard (556.2771 m/z).

## Synthesis of Amino-DBE Compounds – His-DBE and Phe-DBE

The synthesis of the amino acid-DBE substrates was followed using the method described by Harding *et al.*[11](#_ENREF_11)^,^[12](#_ENREF_12)

1. **Synthesis of Boc-aa-DBE**

N-Boc-L-amino acid was added to a 25 mL flask (1.97 mmol) with 3,5-dinitrobenzyl chloride (1.65 mmol). The reaction was placed under argon and DMF (0.4 mL) was added, forming a yellow solution. Et_3_N (3.98 mmol) was added last, and the reaction was stirred overnight at room temperature. The mixture was diluted in diethyl ether (25mL) and an extraction was performed using 1M HCl (2 x 25mL) followed by sat. NaHCO_3_ (2 x 25mL). A brine wash was performed, and the resultant organic layer was dried over MgSO_4_. The filtered solution was concentrated *in vacuo* to yield an orange oil.

1. **Boc deprotection**

Crude N-Boc-amino acid-3,5-dinitrobenzyl ester (aa-DBE) was added to a 25 mL flask whereupon 4 N HCl in 1,4-dioxane (2.5 mL) were added. The resulting solution was stirred at room temperature for 1 hour and the solvent removed in vacuo. The resulting oil was triturated with diethyl ether to yield the final compound.

His-DBE: ^1^H NMR (400 MHz, Methanol-d_4_) δ 8.93 (t, J = 2.0 Hz, 1H), 8.85 (s, 1H), 8.59 (d, J = 2.0 Hz, 2H), 7.49 (s, 1H), 5.48 (d, J = 6.4 Hz, 2H), 4.57 (t, J = 6.8 Hz, 1H), 3.54 – 3.35 (m, 2H).

**Supplementary Fig. 4** NMR spectra of His-DBE

Phe-DBE: ^1^H NMR (400 MHz, Methanol-*d*_4_) δ 8.96 (t, *J* = 2.1 Hz, 1H), 8.51 (d, 2H), 7.30 – 7.19 (m, 5H), 5.41 (s, 2H), 4.45 (t, *J* = 7.2 Hz, 1H), 3.23 (dd, *J* = 7.2, 2.5 Hz, 2H).

**Supplementary Fig. 5** NMR spectra of Phe-DBE

## Trapped acyl-enzyme intermediate

Acyl-intermediate enzyme preparation was performed by incubating 20 mM enzyme with excess aa-DBE (5 mM) overnight. The samples were then sent for analysis at the University of St Andrews mass spectrometry and proteomics facility. The sample was diluted to 1 µM and 10 µL was injected onto a Waters MassPrep column cartridge on a Waters G2TOF LC-MS system. The system used a two solvent system composed of solvent A - Water + 1% F.A:Acetonitrile (95:5) and solvent B - Water + 1% F.A.:Acetonitrile (5:95). A gradient was employed from 2% B to 98% B over 4 minutes, held for 0.5 minutes then returned to 2% B. MS data was collected in ESI+ mode and scanned from 500–2500 m/z. The raw data was combined and processed to mass using Waters MaxEnt algorithm at 0.1 resolution using peak width of half height of 0.4 Da. The instrument was calibrated externally against a solution of horse heart myoglobin, and an internal lock mass of LeuEnk was additionally used.


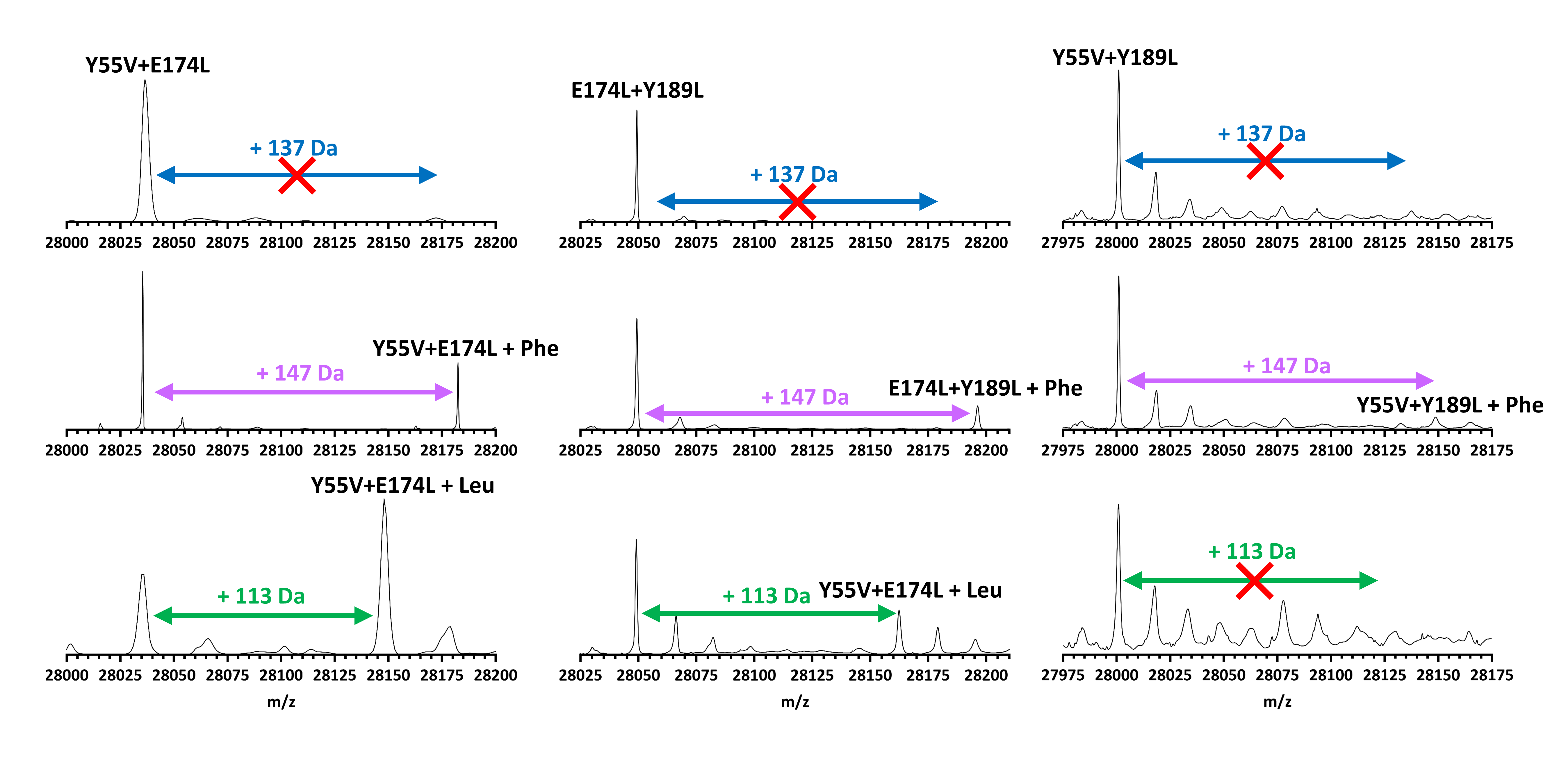


**Supplementary Fig. 6** Intact protein mass spectrometry of mutants incubated with different aa-DBE substrates: histidine, phenylalanine and leucine. None of the mutants are able to react with his-DBE and instead accept phenylalanine and leucine (except Y55V+Y189L which accepts phenylalanine exclusively).

# **Supplementary Note 1 - Mass Spectrometry of Unnatural CDP Library**

| **Amino acid 1** | **Amino acid 2** | **Present?** |
| --- | --- | --- |
| Histidine | Phenylalanine | Yes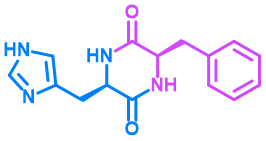 |
| β-(1,2,4-Triazol-3-yl)-DL-alanine | Phenylalanine | Yes 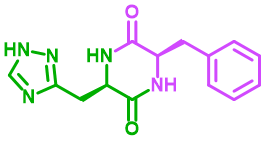 |
| H-β-(2-Thiazolyl)-DL-Ala-OH | Phenylalanine | Yes 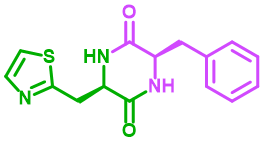 |
| 3-(2-pyridyl)-L-alanine | Phenylalanine | Yes 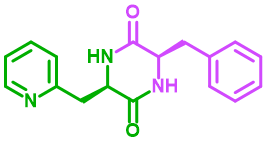 |
| 3-(2-thienyl)-L-Ala-OH | Phenylalanine | Yes 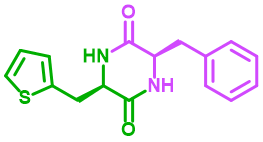 |
| 1-methyl-L-histidine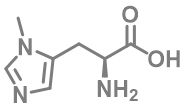 | Phenylalanine | No |
| 3-Methyl-L-histidine 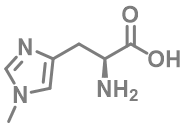 | Phenylalanine | No |
| 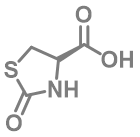L-2-Oxothiazolidine-4-carboxylic acid | Phenylalanine | No |
| 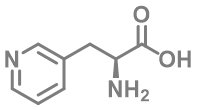3-(3-pyridyl)-L-alanine | Phenylalanine | No |
| 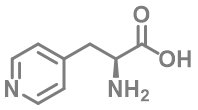3-(4-pyridyl)-L-alanine | Phenylalanine | No |
| Histidine | 4-chloro-L-phenylalanine | Yes 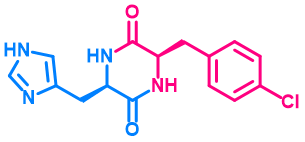 |
| Histidine | 4-bromo-L-phenylalanine | Yes 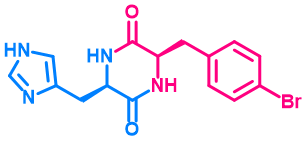 |
| Histidine | 4-Ethynyl-L-phenylalanine | Yes 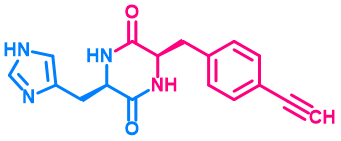 |
| Histidine | 4-Cyano-L-phenylalanine | Yes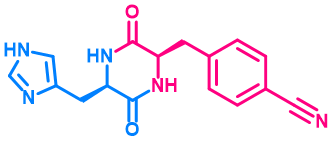 |
| Histidine | 3-(2-thienyl)-L-Ala-OH | Yes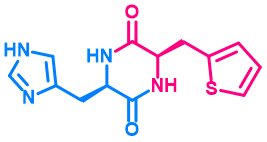 |
| Histidine | 4-azido-L-phenylalanine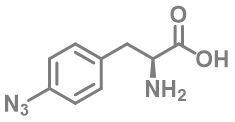 | No |
| Histidine | 4-nitro-L-phenylalanine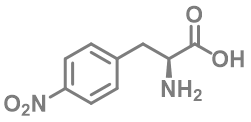 | No |
| Histidine | 4-amino-L-phenylalanine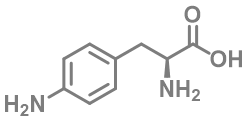 | No |

**Supplementary Table 1.** All non-canonical amino acids tested with *Para*CDPS in individual assays are listed here and rows in green highlight the accepted substrates.

| **Amino acid 1** | **Amino acid 2** | **Present?** |
| --- | --- | --- |
| Histidine | Proline | 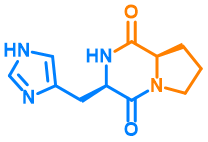Yes |
| β-(1,2,4-Triazol-3-yl)-DL-alanine | Proline | 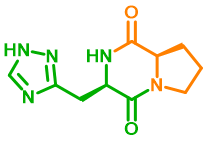Yes |
| H-β-(2-Thiazolyl)-DL-Ala-OH | Proline | 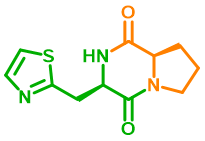Yes |
| 3-(2-pyridyl)-L-alanine | Proline | 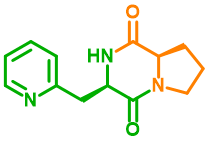Yes |
| 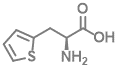3-(2-thienyl)-L-Ala-OH | Proline | No |
| 1-methyl-L-histidine 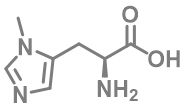 | Proline | No |
| 3-Methyl-L-histidine 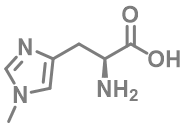 | Proline | No |
| L-2-Oxothiazolidine-4-carboxylic acid 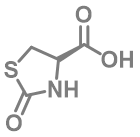 | Proline | No |
| 3-(3-pyridyl)-L-alanine 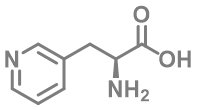 | Proline | No |
| 3-(4-pyridyl)-L-alanine 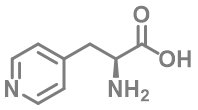 | Proline | No |
| Histidine | Cis-4-Fluoro-L-proline | 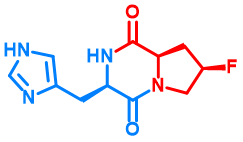Yes |
| Histidine | Trans-4-Fluoro-L-proline | 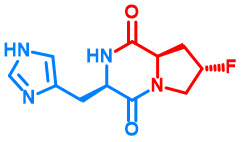Yes |
| Histidine | (S)-4,4-Difluoropyrrolidine-2-carboxylic acid | 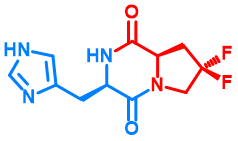Yes |
| Histidine | H-3,4-Dehydro-L-Pro-OH | 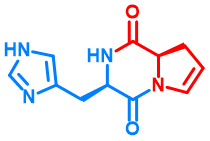Yes |
| Histidine | (2S,4S)-4-Bromopyrrolidine-2-carboxylic acid | 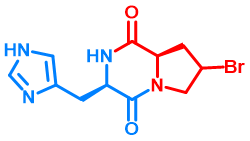Yes |
| Histidine | (R)-α-Propynyl-L-proline Hydrochloride 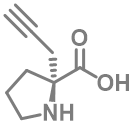 | No |
| Histidine | 4-Amino-cis-L-proline Dihydrochloride 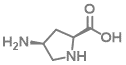 | No |
| Histidine | (2S,4S)-4-Hydroxypyrrolidine-2-carboxylic acid 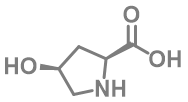 | No |
| Histidine | (2R,4S)-4-Hydroxypyrrolidine-2-carboxylic acid 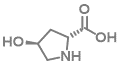 | No |
| Histidine | H-Hyp-OH (trans-hydroxy-L-Pro) 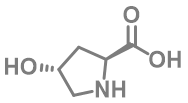 | No |
| Histidine | cis-4-hydroxy-D-proline 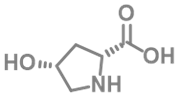 | No |
| Histidine | Glutamate | 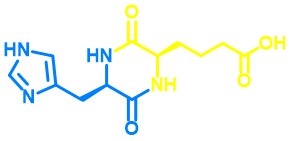Yes |
| Histidine | Quisqualic acid 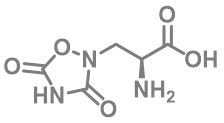 | No |
| Histidine | Ibotenic acid 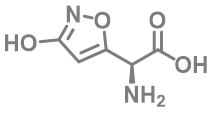 | No |
| Histidine | α-amino-3-hydroxy-5-methyl-4-isoxazolepropionic acid  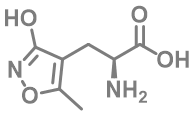 | No |

**Supplementary Table 2.** All non-canonical amino acids tested with *Parcu*CDPS in individual assays are listed here and rows in green highlight the accepted substrates.

**
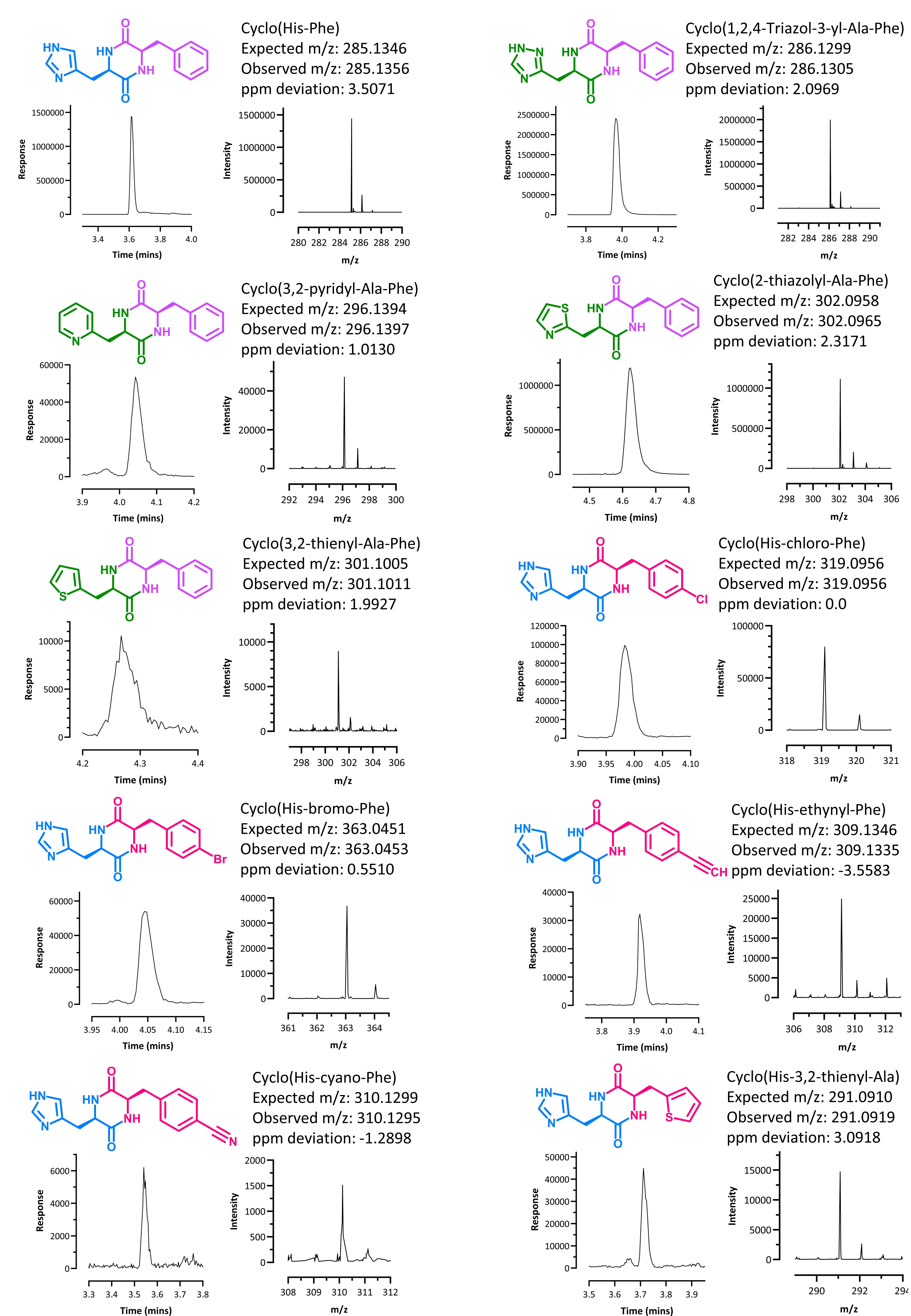
**

**Supplementary Fig. 7** Mass spectrometry of the products formed by *Para*CDPS using a combination of canonical and non-canonical amino acids.


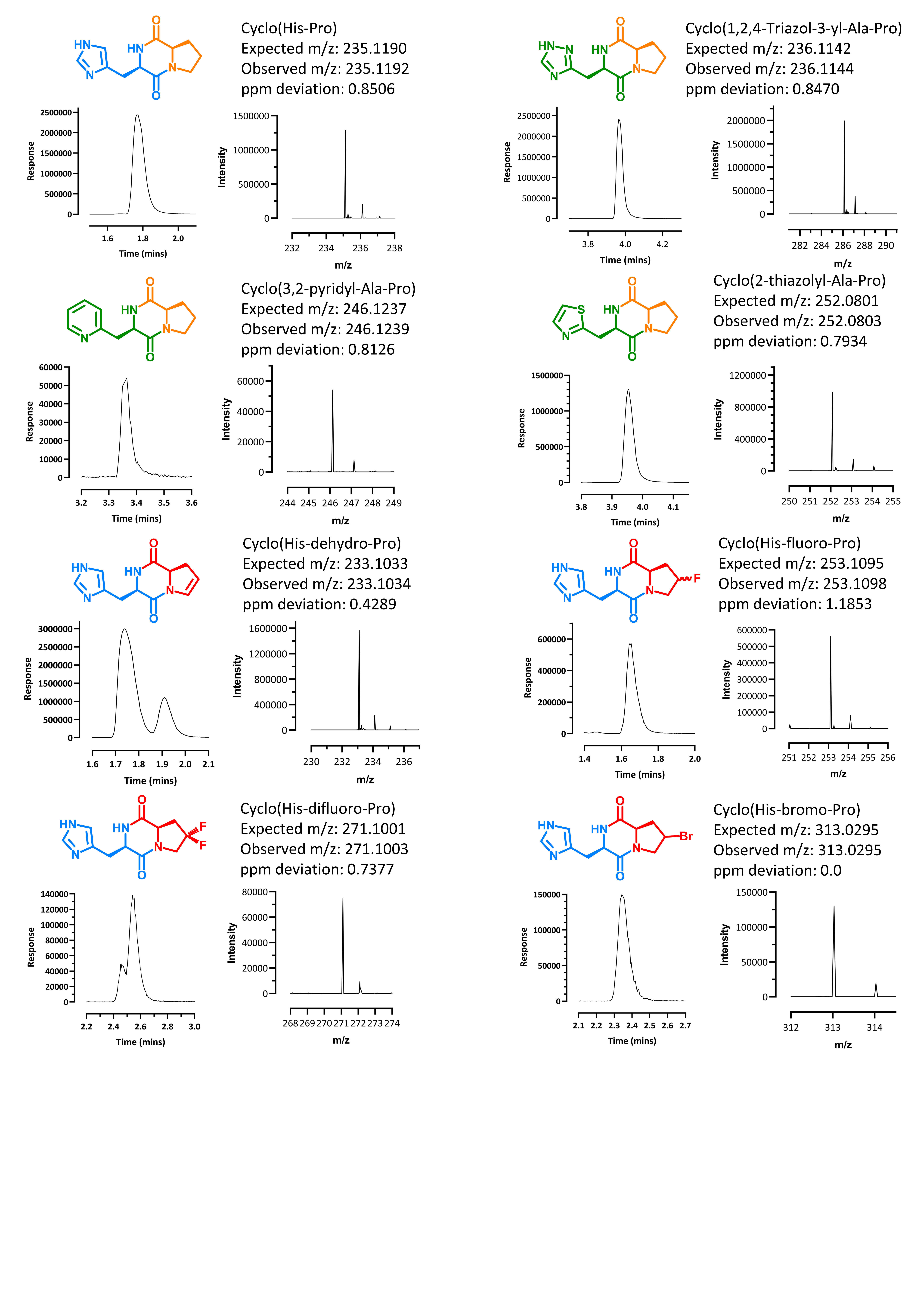
**Supplementary Fig. 8** Mass spectrometry of the products formed by *Parcu*CDPS using a combination of canonical and non-canonical amino acids.


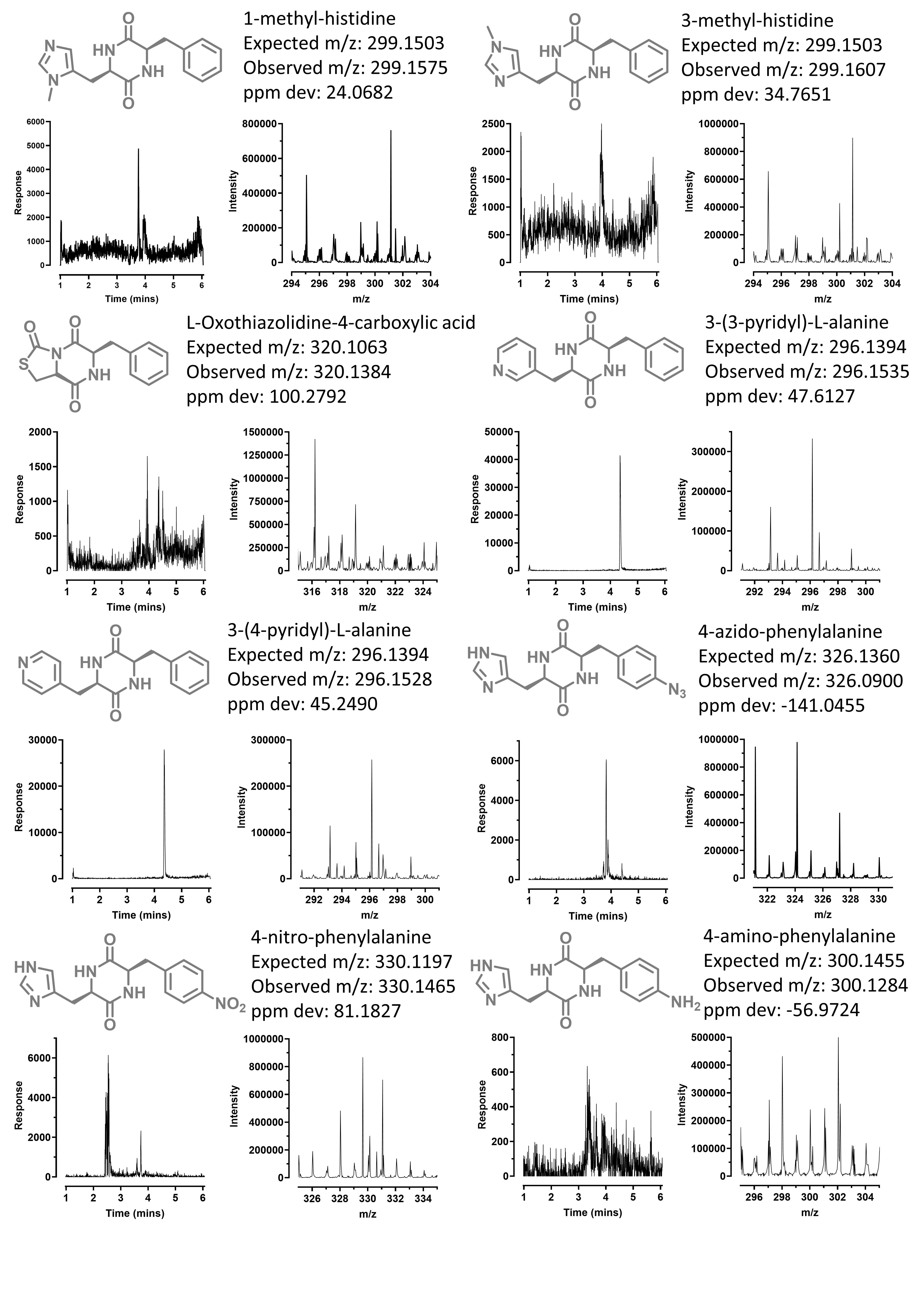


**Supplementary Fig. 9** Mass spectrometry of products **not** formed by *Para*CDPS using a combination of canonical and non-canonical amino acids. Expected products drawn in grey alongside EICs and mass chromatograms to confirm the lack of product.


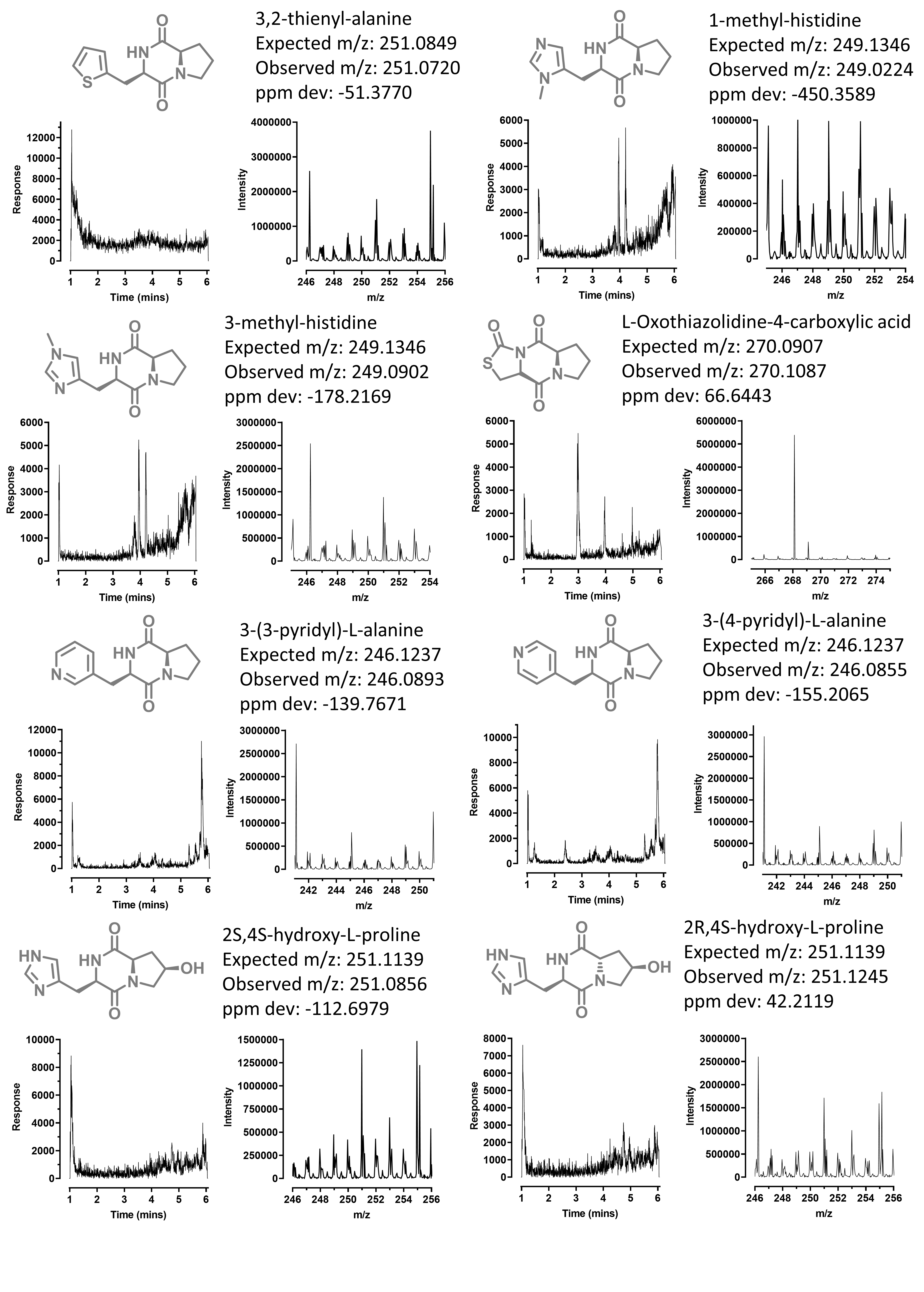

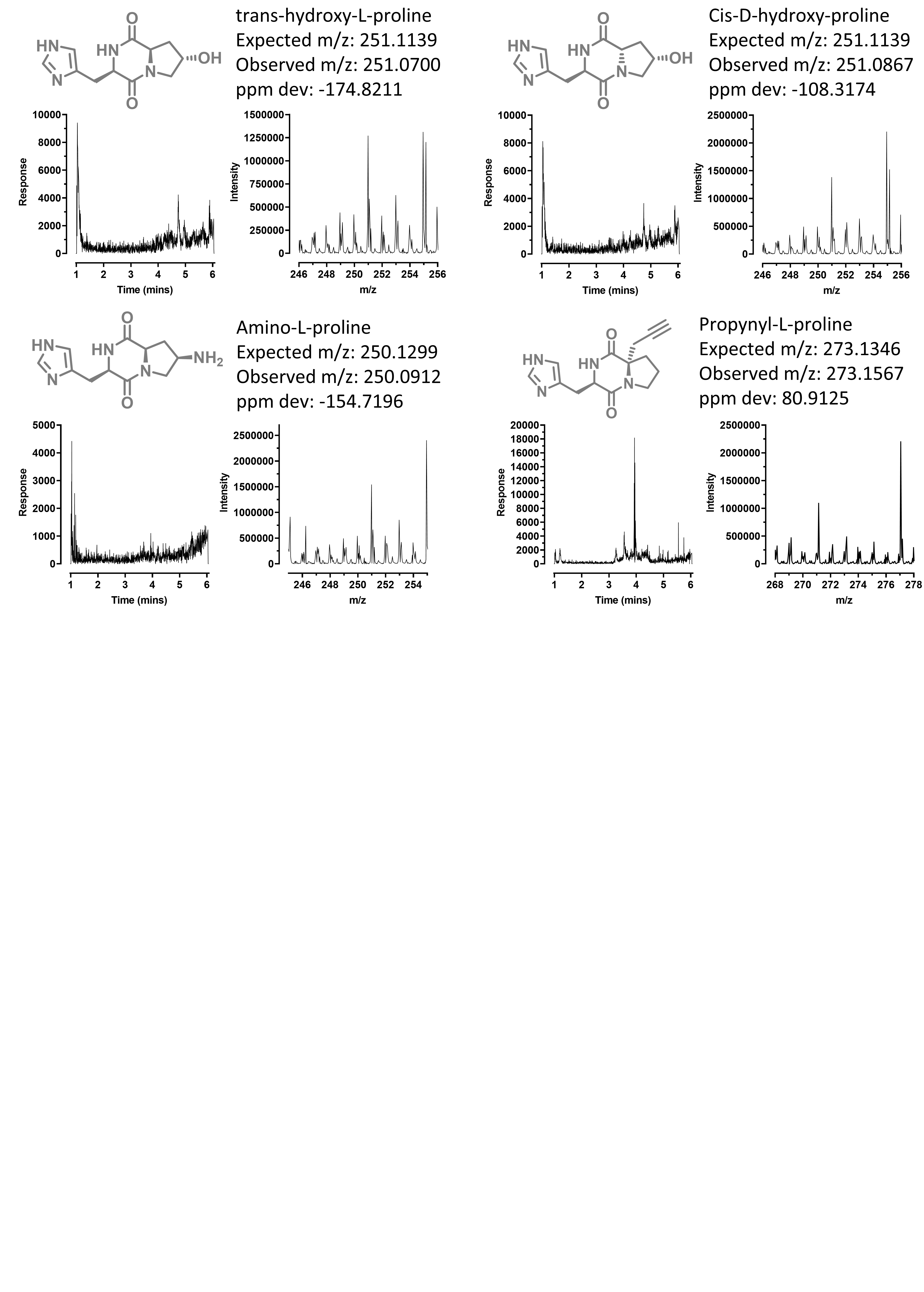


**Supplementary Fig. 10** Mass spectrometry of products **not** formed by *Parcu*CDPS using a combination of canonical and non-canonical amino acids.

# **Supplementary Note 2 - HPLC Analysis of cHE and cHP**


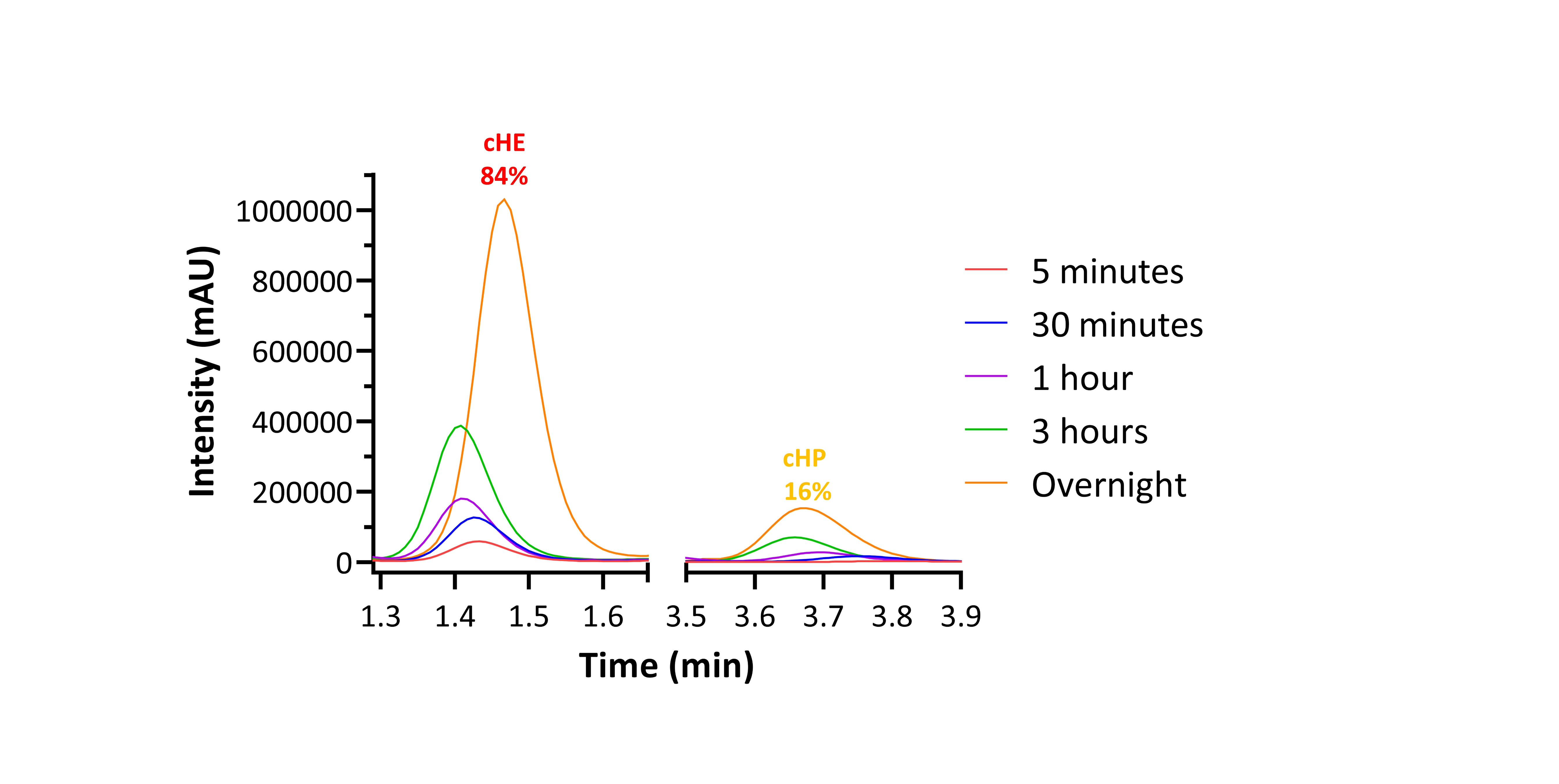
A time course experiment was performed using a reaction mixture composed of equal concentrations of proline and glutamate with *Parcu*CDPS. Reaction was quenched at different time points – 5 minutes, 30 minutes, 1 hour, 3 hour and overnight – using the methanol method previously described. High performance liquid chromatography (HPLC) of each sample was performed using a Shimadzu HPLC system coupled to a UV-vis detector equipped with a Waters XSelect Premier HSS T3 column (2.5 µm, 4.6mm x 50mm). The column was heated to 40 °C for the run and 25 µL of sample was injected per run. Mobile phases were A - water + 0.1% trifluoroacetic acid, and B – 100% acetonitrile. The analytes were separated using a gradient mobile phase from 1% B to 50% B over 5 minutes using a flow rate of 1 mL min^-1^ and the absorbance at 214 nm and 254 nm was monitored.

**Supplementary Fig. 11** HPLC chromatogram of reaction mixture at 214 nm.

# **Supplementary Note 3 - Superimposition of *Parcu*CDPS with most similar known CDPS**


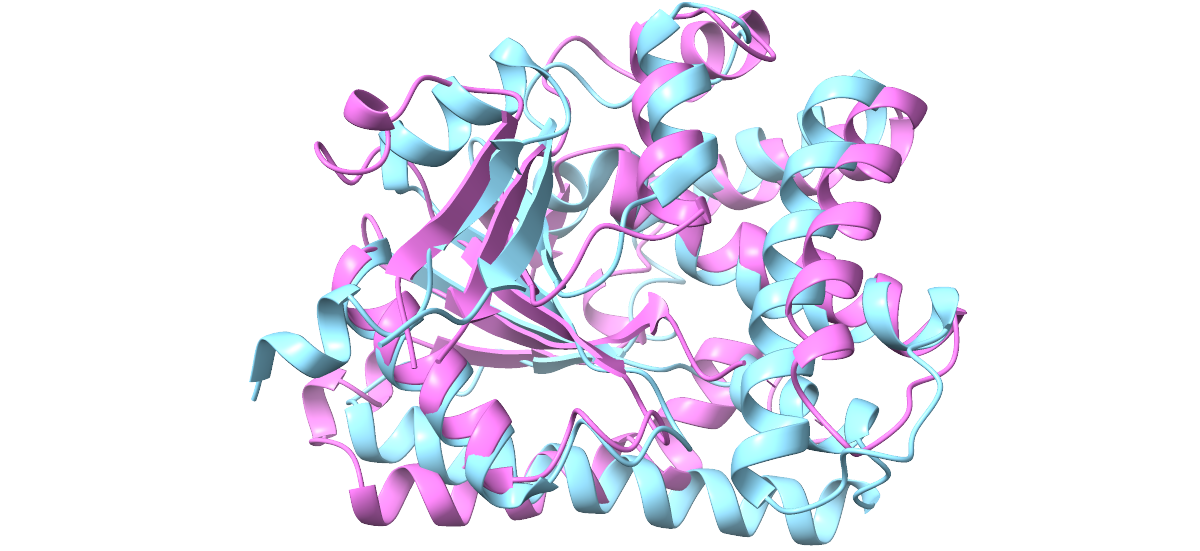


**Supplementary Fig. 12** Superimposition of *Parcu*CDPS WT (pink) with the most similar CDPS - BtCDPS (blue, 6ZTU). Structures manipulated using SSM Superpose on WinCoot where the core rmsd was calculated: 2.7464 Å over 174 aligned residues.

# **Supplementary Note 4 - *Parcu*CDPS Mutant Crystal Structures**

**
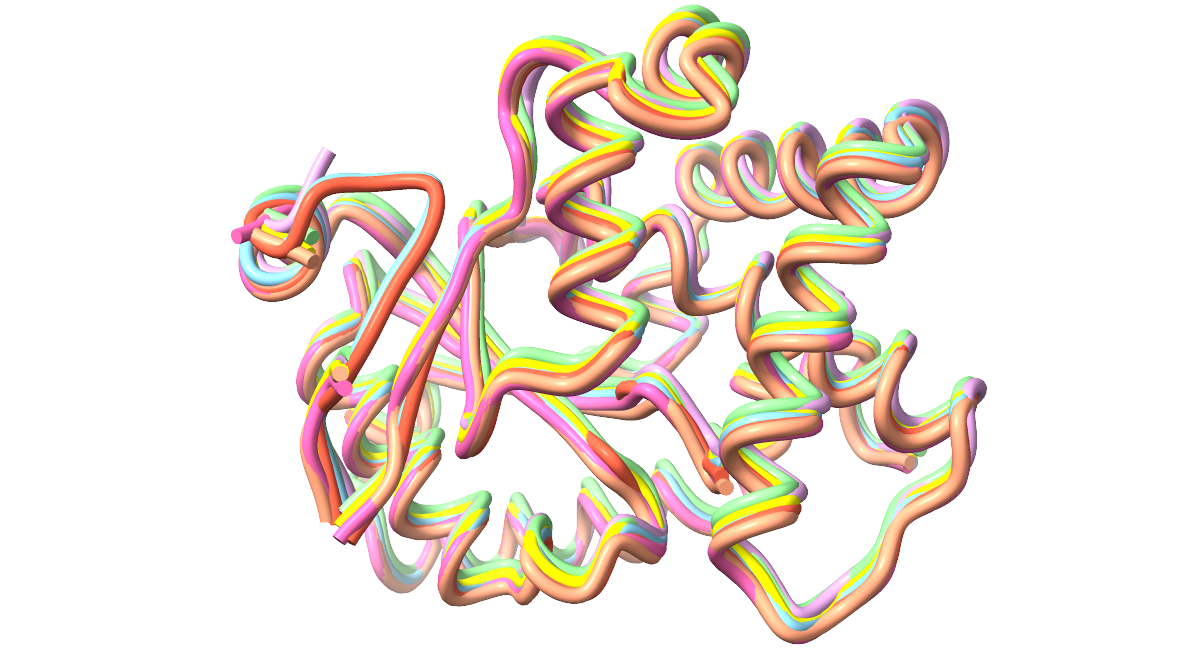
**

**Supplementary Fig. 13** Superimposition of *Parcu*CDPS WT and the 6 crystallised mutant structures: WT (blue); Y55F (green); D58N (pink); E171Q (orange); E174A (yellow); E174L (beige) and Y189F (lilac).

# **Supplementary Note 5 - *Parcu*CDPS Crystallographic Data Table**

|  | ***Parcu*CDPS WT** | ***Parcu*CDPS Y55F** | ***Parcu*CDPS D58N** | ***Parcu*CDPS E171Q** | ***Parcu*CDPS E174A** | ***Parcu*CDPS E174L** | ***Parcu*CDPS Y189F** |
| --- | --- | --- | --- | --- | --- | --- | --- |
| Accession code | 7QB8 | 7QAY | 7QAU | 7QAX | 7QAQ | 7QAT | 7QAW |
| Resolution (Å) | 51.31 - 1.90 (1.97 - 1.90) | 45.28 - 2.09 (2.12 - 2.09) | 46.26 - 2.30 (2.38 - 2.30) | 46.05 - 2.09 (2.16 - 2.09) | 44.85 - 2.40 (2.49 - 2.40) | 73.29 – 2.40 (2.44 – 2.4) | 45.26 - 2.29 (2.37 - 2.29) |
| Space group | P 42 21 2 | P 42 21 2 | P 42 21 2 | P 42 21 2 | P 42 21 2 | P 42 21 2 | P 42 21 2 |
| Cell Dimensions a, b, c (Å) | 102.61, 102.61, 49.76 | 101.26, 101.26, 50.07 | 103.44, 103.44, 49.95 | 102.97, 102.97, 49.81 | 102.60, 102.60, 49.86 | 103.63, 103.63, 49.68 | 101.20, 101.20, 50.32 |
| α, β, γ (°) | 90, 90, 90 | 90, 90, 90 | 90, 90, 90 | 90, 90, 90 | 90, 90, 90 | 90, 90, 90 | 90, 90, 90 |
| Total reflections | 1128270 (101638) | 192351 (6030) | 311233 (30217) | 222211 (4134) | 260149 (25838) | 282452 (11766) | 187130 (8625) |
| Unique reflections | 21493 (2107) | 14927 (1536) | 12408 (1189) | 15221 (1565) | 10887 (1064) | 11063 (534) | 12240 (1215) |
| Multiplicity | 52.5 (48.2) | 12.9 (7.9) | 25.1 (25.4) | 14.5 (5.5) | 23.9 (24.3) | 25.5 (22) | 15.3 (14.5) |
| Completeness (%) | 99.86 (99.86) | 94.1 (100.0) | 98.92 (97.62) | 92.57 (96.84) | 99.94 (99.91) | 100 (100) | 99.12 (99.92) |
| Mean I/sigma(I) | 31.45 (1.95) | 94.02 (99.81) | 27.09 (4.21) | 15.80 (0.90) | 26.17 (7.24) | 26.00 (0.70) | 18.80 (1.30) |
| R-merge | 0.073 (1.452) | 0.069 (1.452) | 0.088 (1.435) | 0.090 (1.637) | 0.155 (0.9867) | 0.055 (0.884) | 0.072 (2.230) |
| R-pim | 0.010 (0.207) | 0.027 (0.804) | 0.018 (0.287) | 0.023 (0.807) | 0.033 (0.202) | 0.011 (0.189) | 0.019 (0.604) |
| CC1/2 | 0.999 (0.926) | 1.000 (0.490) | 1.000 (0.892) | 0.999 (0.331) | 0.999 (0.963) | 1.000 (0.947) | 1.000 (0.511) |
| R-work | 0.2248 | 0.2108 | 0.2096 | 0.2260 | 0.1955 | 0.2139 | 0.2187 |
| R-free | 0.2419 | 0.2625 | 0.2473 | 0.2647 | 0.2451 | 0.2606 | 0.2475 |
| Total non-hydrogen atoms | 1910 | 1828 | 1825 | 1885 | 1834 | 1817 | 1829 |
| Total macromolecules | 1870 | 1813 | 1825 | 1870 | 1810 | 1809 | 1820 |
| Total ligands | 0 | 0 | 0 | 0 | 0 | 0 | 0 |
| Total solvent | 40 | 15 | 11 | 15 | 24 | 8 | 9 |
| Total protein residues | 221 | 215 | 215 | 221 | 215 | 215 | 216 |
| RMS(bonds) (Å) | 0.008 | 0.008 | 0.007 | 0.008 | 0.009 | 0.008 | 0.008 |
| RMS(angles) (°) | 1.15 | 0.93 | 1.10 | 1.15 | 1.21 | 0.90 | 0.88 |
| Ramachandran favored (%) | 98.62 | 96.65 | 97.61 | 97.24 | 97.61 | 96.65 | 97.62 |
| Ramachandran allowed (%) | 1.38 | 2.87 | 1.91 | 2.30 | 1.91 | 2.87 | 1.90 |
| Ramachandran outliers (%) | 0.0 | 0.48 | 0.48 | 0.46 | 0.48 | 0.48 | 0.48 |
| Average B-factor (Å^2^) | 63.41 | 71.32 | 67.79 | 68.15 | 55.38 | 90.10 | 85.06 |

**Supplementary Table 3**. Crystallographic data table for *Parcu*CDPS and its corresponding variants.

# **Supplementary Note 6 - Comparison of mutant pKa values**

**Supplementary Table 4. pKa values calculated using PROPKA.**[1](#_ENREF_1) Model pKa refers to expected pKas for the side chain of each amino acid free in solution, while pKa is the calculated number for each residue using the pdb file for each variant.

| WT | | |  |
| --- | --- | --- | --- |
| Residue | pKa | model-pKa |  |
| D58 | 6.32 | 3.8 |  |
| E171 | 10.51 | 4.5 |  |
| E174 | 4.96 | 4.5 |  |
| Y55 | 18.05 | 10 |  |
| Y167 | 9.89 | 10 |  |
| Y189 | 14.12 | 10 |  |
| Y191 | 13.19 | 10 |  |

| D58N | | |  | Y55F | | |
| --- | --- | --- | --- | --- | --- | --- |
| Residue | pKa | model-pKa |  | Residue | pKa | model-pKa |
| D58 |  |  |  | D58 | 6.18 | 3.8 |
| E171 | 9.32 | 4.5 |  | E171 | 11.36 | 4.5 |
| E174 | 4.74 | 4.5 |  | E174 | 8.89 | 4.5 |
| Y55 | 17.93 | 10 |  | Y55 |  |  |
| Y167 | 9.8 | 10 |  | Y167 | 9.75 | 10 |
| Y189 | 13.89 | 10 |  | Y189 | 13.13 | 10 |
| Y191 | 11.78 | 10 |  | Y191 | 12.84 | 10 |

| E174L | | | |  | | E174A | | |  |
| --- | --- | --- | --- | --- | --- | --- | --- | --- | --- |
| Residue | pKa | model-pKa |  | | Residue | | pKa | model-pKa |  |
| D58 | 5.59 | 3.8 |  | | D58 | | 6.03 | 3.8 |  |
| E171 | 9.95 | 4.5 |  | | E171 | | 10.09 | 4.5 |  |
| E174 |  |  |  | | E174 | |  |  |  |
| Y55 | 14.49 | 10 |  | | Y55 | | 14.08 | 10 |  |
| Y167 | 9.54 | 10 |  | | Y167 | | 9.57 | 10 |  |
| Y189 | 13.16 | 10 |  | | Y189 | | 13.25 | 10 |  |
| Y191 | 12.72 | 10 |  | | Y191 | | 12.68 | 10 |  |

| E171Q | | |  | Y189F | | |
| --- | --- | --- | --- | --- | --- | --- |
| Residue | pKa | model-pKa |  | Residue | pKa | model-pKa |
| D58 | 6.11 | 3.8 |  | D58 | 5.61 | 3.8 |
| E171 |  |  |  | E171 | 10.83 | 4.5 |
| E174 | 5.78 | 4.5 |  | E174 | 7.73 | 4.5 |
| Y55 | 18.28 | 10 |  | Y55 | 16.49 | 10 |
| Y167 | 9.85 | 10 |  | Y167 | 9.41 | 10 |
| Y189 | 14.21 | 10 |  | Y189 |  |  |
| Y191 | 13.13 | 10 |  | Y191 | 11.72 | 10 |

# **Supplementary Note 7 -** **Differential scanning fluorimetry of *Parcu*CDPS mutants**


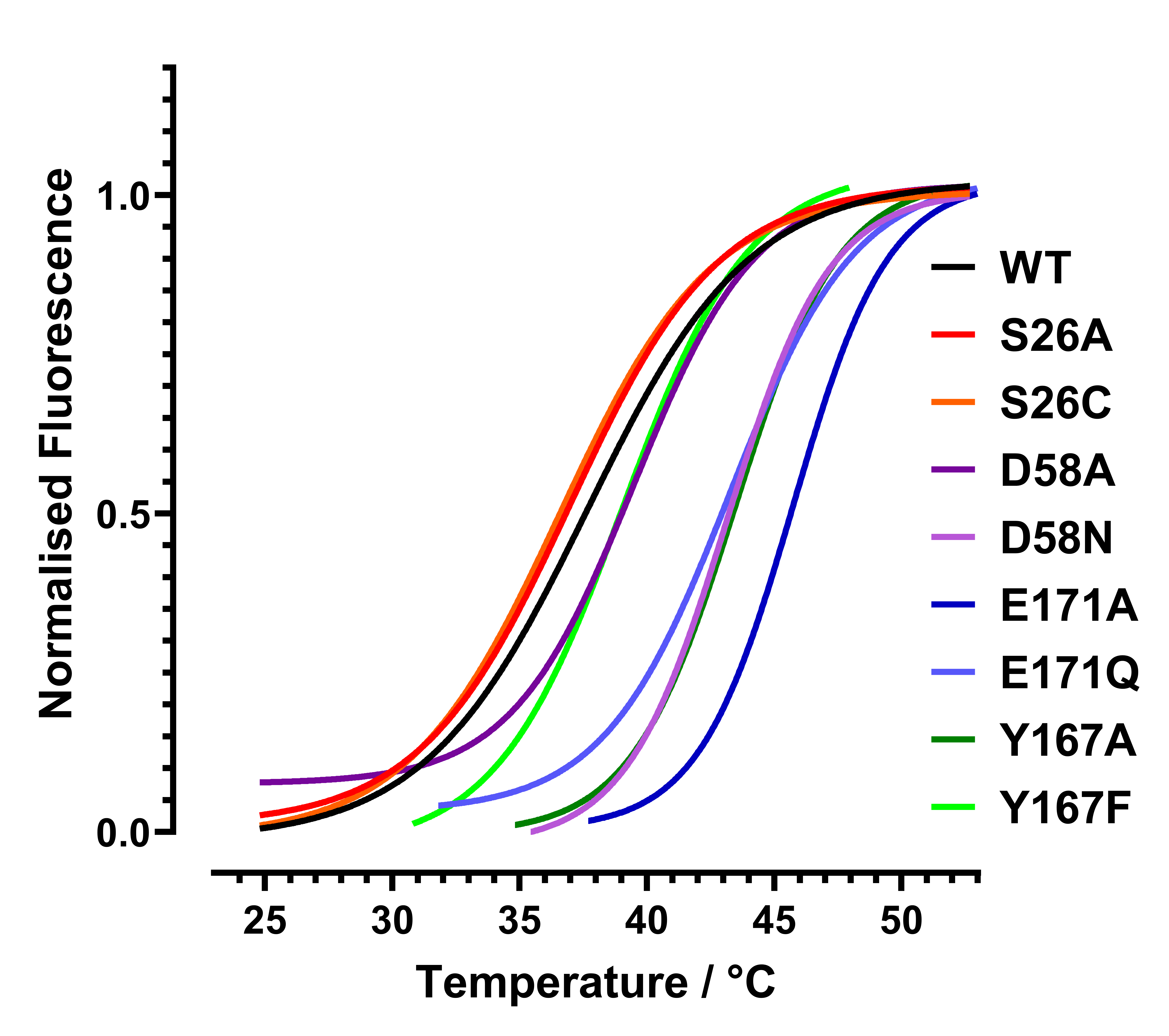


**Supplementary Fig. 14A** Differential scanning fluorimetry of *Parcu*CDPS and mutants from the active site residues.. Each line represents a sigmoidal fit of the normalised mean of 3 replicates for each protein. The melting temperatures were recorded: 37.65°C (WT); 36.98°C (S26A); 36.58°C (S26C); 39.50°C (D58A); 43.17°C (D58N); 45.75°C (E171A); 43.25°C (E171Q); 43.47°C (Y167A) and 39.04°C (Y167F)


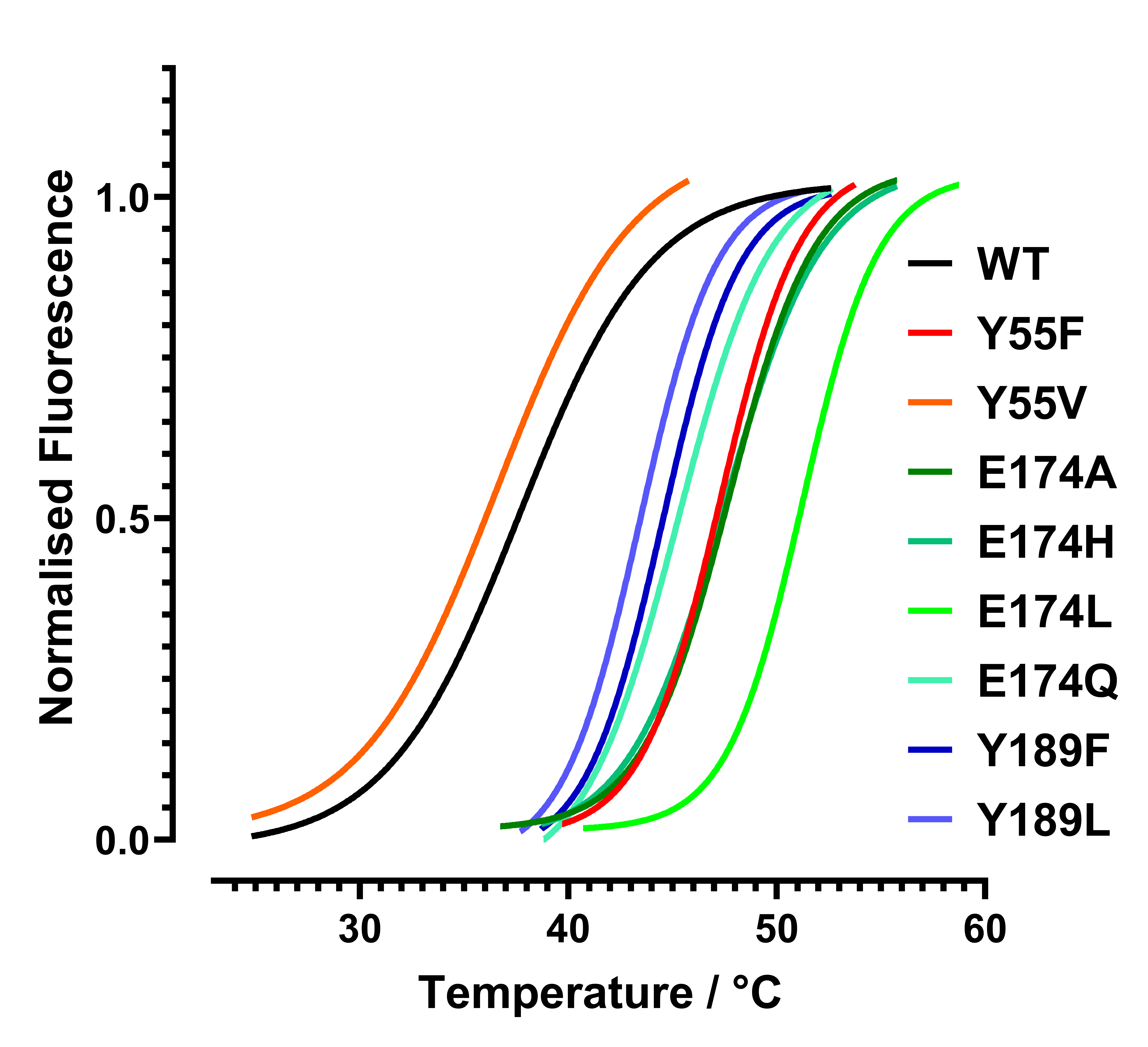


**Supplementary Fig. 14B** Differential scanning fluorimetry of *Parcu*CDPS and mutants of pocket 1 residues. Each line represents a sigmoidal fit of the normalised mean of 3 replicates for each protein. The melting temperatures were recorded: 37.65°C (WT); 47.31°C (Y55F); 36.59°C (Y55V); 47.70°C (E174A); 47.53°C (E174H); 51.25°C (E174L); 45.25°C (E174Q); 44.54°C (Y189F) and 43.44°C (Y189L).

**
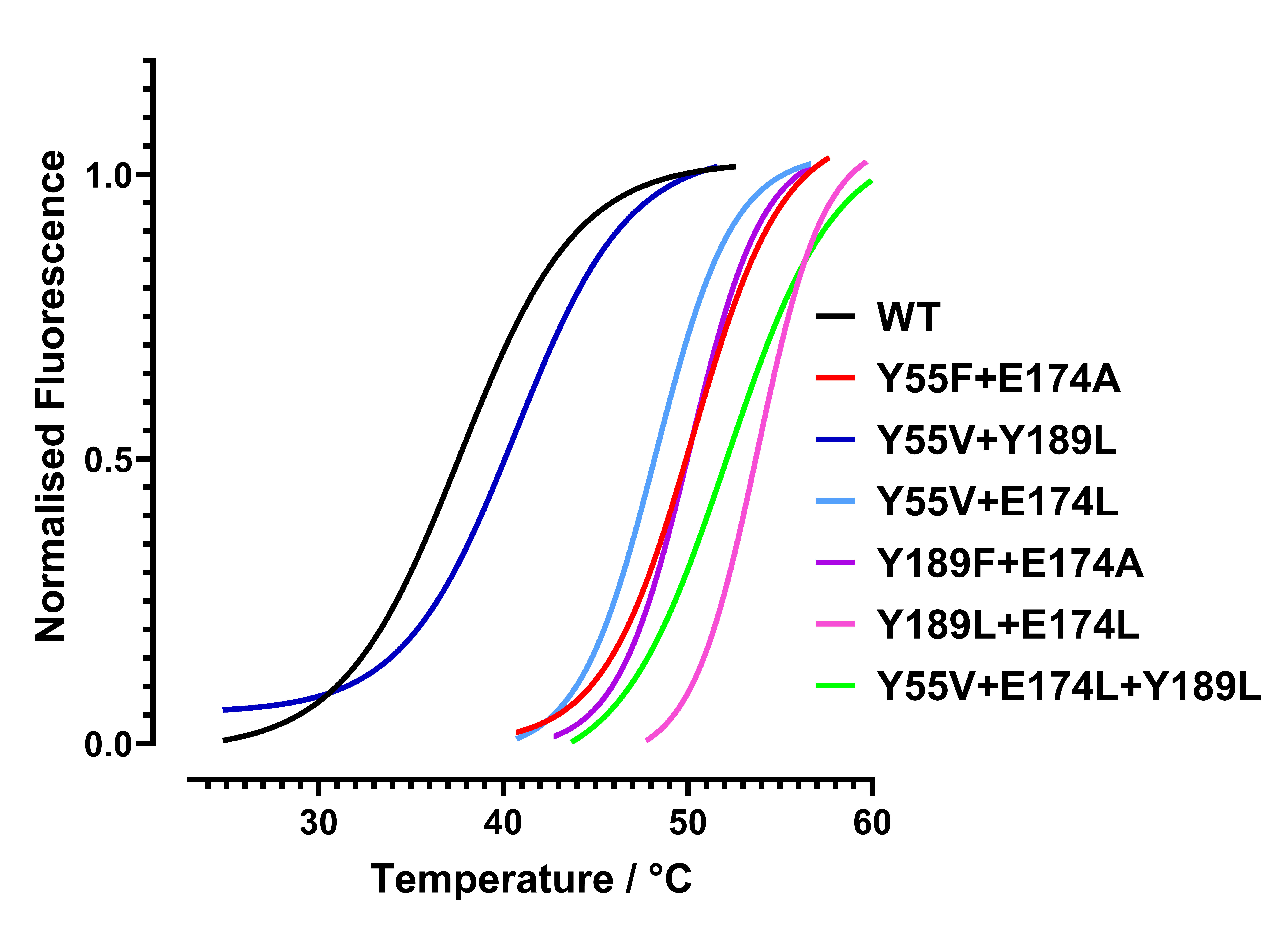
**

**Supplementary Fig. 14C** Differential scanning fluorimetry of *Parcu*CDPS and double and triple mutant combinations of P1 residues. Each line represents a sigmoidal fit of the normalised mean of 3 replicates for each protein. The melting temperatures were recorded: 37.65°C (WT); 50.27°C (Y55F+E174A); 40.68°C (Y55V+Y189L); 48.27°C (Y55V+E174L); 50.12°C (Y189F+E174A); 53.79°C (Y189L+E174L) and 52.14°C (Y55V+E174L+Y189L).

# **Supplementary Note 8 - Histidine binding pocket comparison**


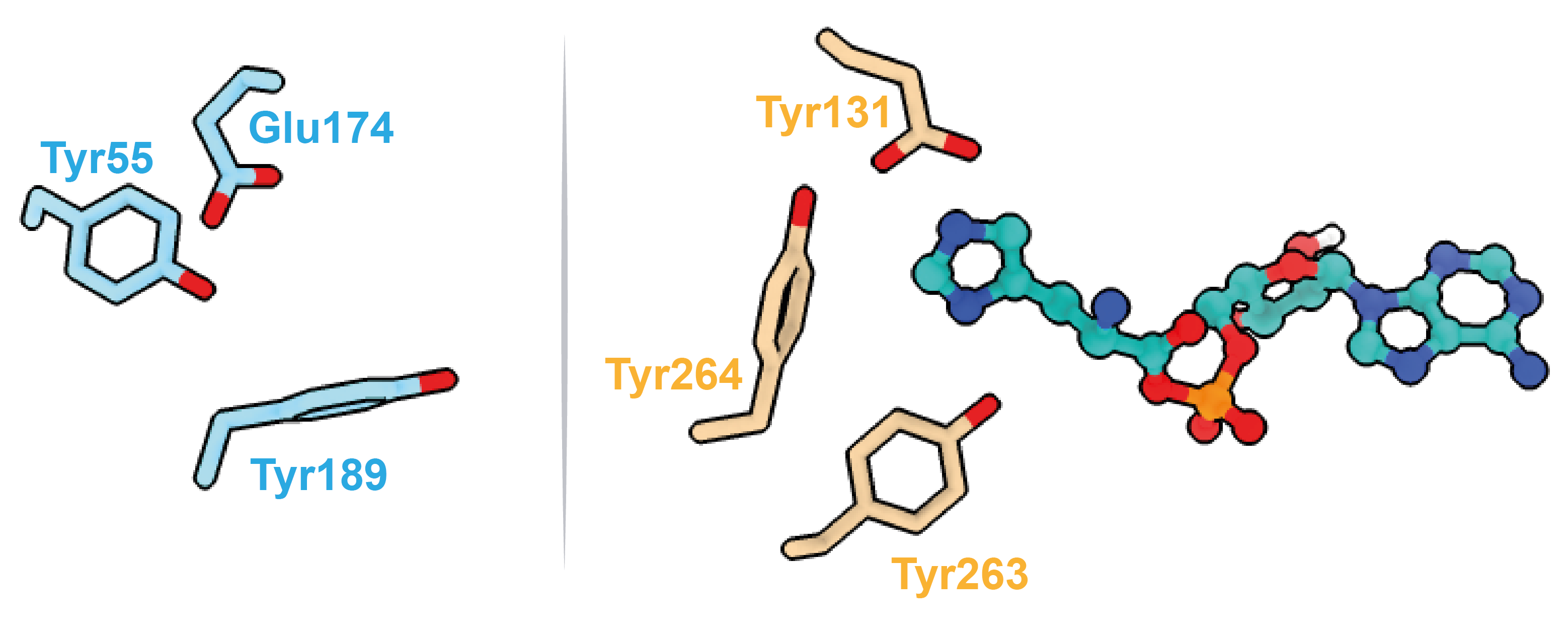


**Supplementary Fig. 15** Comparison between histidine binding pockets of HisRS (beige, pdb 1kmm, bound to histidinyl-adenylate intermediate) and *Parcu*CDPS (cyan).

# **Supplementary Note 9 - Novel cyclodipeptides**

| **Amino acid 1** | **Amino acid 2** | **Present on Reaxys?** |
| --- | --- | --- |
| Histidine | Phenylalanine | Yes |
| β-(1,2,4-Triazol-3-yl)-DL-alanine | Phenylalanine | No |
| H-β-(2-Thiazolyl)-Ala-OH | Phenylalanine | No |
| 3-(2-pyridyl)-L-alanine | Phenylalanine | No - meta isomer present[13](#_ENREF_13) |
| 3-(2-thienyl)-L-Ala-OH | Phenylalanine | No |
| Histidine | 4-chloro-L-phenylalanine | No |
| Histidine | 4-bromo-L-phenylalanine | No |
| Histidine | 4-Ethynyl-L-phenylalanine | No |
| Histidine | 4-Cyano-L-phenylalanine | Yes - patent |
| Histidine | 3-(2-thienyl)-L-Ala-OH | Yes |
| Histidine | Proline | Yes |
| β-(1,2,4-Triazol-3-yl)-DL-alanine | Proline | No |
| H-β-(2-Thiazolyl)-Ala-OH | Proline | No |
| 3-(2-pyridyl)-L-alanine | Proline | No |
| Histidine | cis-fluoro-Pro | No |
| Histidine | trans-fluoro-Pro | No |
| Histidine | (S)-4,4-Difluoropyrrolidine-2-carboxylic acid | No |
| Histidine | H-3,4-Dehydro-Pro-OH | No |
| Histidine | (2S,4S)-4-Bromopyrrolidine-2-carboxylic acid | No |
| Histidine | Glutamate | Yes |

**Supplementary Table 5**. Novel molecules produced here. Reaxys refers to searches on the server <https://www.reaxys.com/#/search/quick>

# **Supplementary References**

1 Jurrus, E. *et al.* Improvements to the APBS biomolecular solvation software suite. *Protein Sci* **27**, 112-128, doi:10.1002/pro.3280 (2018).

2 Gibson, D. G. *et al.* Enzymatic assembly of DNA molecules up to several hundred kilobases. *Nature Methods* **6**, 343-345, doi:10.1038/nmeth.1318 (2009).

3 Liebschner, D. *et al.* Macromolecular structure determination using X-rays, neutrons and electrons: recent developments in Phenix. *Acta Crystallographica Section D* **75**, 861-877, doi:doi:10.1107/S2059798319011471 (2019).

4 Emsley, P., Lohkamp, B., Scott, W. G. & Cowtan, K. Features and development of Coot. *Acta Crystallogr D Biol Crystallogr* **66**, 486-501, doi:10.1107/S0907444910007493 (2010).

5 Joosten, R. P. *et al.* PDB_REDO: automated re-refinement of X-ray structure models in the PDB. *J Appl Crystallogr* **42**, 376-384, doi:10.1107/S0021889809008784 (2009).

6 Bertand Beckert, B. M. Synthesis of RNA by In Vitro Transcription. *RNA: Methods in molecular biology* **703**, 29-41 (2011).

7 Mechulam, Y., Guillon, L., Yatime, L., Blanquet, S. & Schmitt, E. in *Methods in Enzymology* Vol. 430 (ed Jon Lorsch) 265-281 (Academic Press, 2007).

8 Hernandez, V. *et al.* Discovery of a novel class of boron-based antibacterials with activity against gram-negative bacteria. *Antimicrob Agents Chemother* **57**, 1394-1403, doi:10.1128/AAC.02058-12 (2013).

9 Shimizu, Y. *et al.* Cell-free translation reconstituted with purified components. *Nat Biotechnol* **19**, 751-755, doi:10.1038/90802 (2001).

10 Krinsky, N. *et al.* A Simple and Rapid Method for Preparing a Cell-Free Bacterial Lysate for Protein Synthesis. *PLOS ONE* **11**, e0165137, doi:10.1371/journal.pone.0165137 (2016).

11 Harding, C. J., Sutherland, E., Hanna, J. G., Houston, D. R. & Czekster, C. M. Bypassing the requirement for aminoacyl-tRNA by a cyclodipeptide synthase enzyme. *RSC Chem. Bio.* **2**, 230-240, doi:10.1039/D0CB00142B (2021).

12 Peacock, J. R. *et al.* Amino acid-dependent stability of the acyl linkage in aminoacyl-tRNA. *RNA* **20**, 758-764, doi:10.1261/rna.044123.113 (2014).

13 Canu, N. *et al.* Incorporation of Non-canonical Amino Acids into 2,5-Diketopiperazines by Cyclodipeptide Synthases. *Angewandte Chemie International Edition* **57**, 3118-3122, doi:10.1002/anie.201712536 (2018).
