# Supplementary material for: Active site remodelling of a cyclodipeptide synthase redefines substrate scope
Source: Commun Chem. 2022 Aug 25;5:101. doi: 10.1038/s42004-022-00715-2 (PMC7613923; doi:10.1038/s42004-022-00715-2)
Supplement: Supplementary file 3 — Description of Additional Supplementary Files [file 42004_2022_715_MOESM3_ESM.docx]

Description of Additional Supplementary Files

**File name:** Supplementary Data 1

**Description:** cif data file for Supplementary Data 1

**File name:** Supplementary Data 2

**Description:** cif data file for Supplementary Data 2

**File name:** Supplementary Data 3

**Description:** cif data file for Supplementary Data 3

**File name:** Supplementary Data 4

**Description:** cif data file for Supplementary Data 4

**File name:** Supplementary Data 5

**Description:** cif data file for Supplementary Data 5

**File name:** Supplementary Data 6

**Description:** cif data file for Supplementary Data 6

**File name:** Supplementary Data 7

**Description:** cif data file for Supplementary Data 7
