# Supplementary material for: Active site remodelling of a cyclodipeptide synthase redefines substrate scope
Source: Commun Chem. 2022 Aug 25;5:101. doi: 10.1038/s42004-022-00715-2 (PMC7613923; doi:10.1038/s42004-022-00715-2)
Supplement: Supplementary file 1 — Czekster_PR File [file 42004_2022_715_MOESM1_ESM.pdf]

nature portfolio

Peer Review File

Active site remodelling of a cyclodipeptide synthase redefines substrate scope

Open Access This file is licensed under a Creative Commons Attribution 4.0 International License, which permits use, sharing, adaptation, distribution and reproduction in any medium or format, as long as you give appropriate credit to the original author(s) and the source, provide a link to the Creative Commons license, and indicate if changes were made. In the cases where the authors are anonymous, such as is the case for the reports of anonymous peer reviewers, author attribution should be to 'Anonymous Referee' followed by a clear attribution to the source work. The images or other third party material in this file are included in the article's Creative Commons license, unless indicated otherwise in a credit line to the material. If material is not included in the article's Creative Commons license and your intended use is not permitted by statutory regulation or exceeds the permitted use, you will need to obtain permission directly from the copyright holder. To view a copy of this license, visit http://creativecommons.org/licenses/by/4.0/.

Reviewers' comments:

Reviewer #1 (Remarks to the Author):

The manuscript by Sutherland and colleagues describes the structural and functional characterization of the cyclodipeptide synthases (CDPS) that use amino acyl-tRNA molecules as activated forms of amino acids that can be used to form diketopiperazine products with different amino acid side chains. The authors determine the structure of the CDPS enzyme from Parcubacterium as well as several mutant enzymes. On the basis of the structure, they design several generations of mutant enzymes and test for the ability to use alternate substrates to produce different CDP products.

The structure determination seems well done with one exception. The diffraction statistics all look quite good. The crystallographic R-factors however all are above 20%. Looking at the number of solvent molecules, which range from 2 to 40 molecules, this may be the cause of the unusually high R-factors. For 1.9Å – 2.3 Å datasets, the authors may wish to see if their density allows for the inclusion of more waters, which may allow the refinements to proceed to completion.

The biochemical experiments are well done. As noted below, the description of experimental methods and rationale for experiments is often minimal and the authors must revise the text to make sure that they are unambiguous or provide sufficient detail to analyze or reproduce these experiments. Additionaly, a few sentences in the Results section that better place the experiments in context will save the reader from needing to read the methods section to understand the experiments.

Finally, some journals have policies about statements of priority ("first example of ..." page 13) and the authors may wish to soften some of their pronouncements that "their work pioneers..." or about their "remarkable shift in substrate specificity" or "diverse library of unprecedented compounds."

Specifically:

Page 1. Paragraph 1 of introduction. The final two sentences are unclear about whether "this work" refers to references 8-10 or refers to the current manuscript.

Page 2. Bottom paragraph. The paper would benefit from an introduction to the mechanism of the CDPS enzymes that more fully describes the P1 and P2 pockets and the acyl-enzyme intermediate. The top of page 7 is the first place in which the authors note that the reaction proceeds through acylation. The roles of the specific pockets are never fully elaborated, only the broader differences of P1 being deeper and more restrictive. Similarly, it seems an oversight to not more clearly describe the existing structure of CDPS enzymes, in particular the AlbC acyl enzyme intermediate, which would place the current effort in proper context.

Page 4. Top paragraph. The last paragraph of Page 3 states that the authors wished to avoid purification of individual AA-tRNA synthetases. The paragraph on page 4 then says that they performed their assay with the tRNA pool and the aaRS of choice. No method for purification of an aaRS is provided. Please clarify the source of the aaRS enzymes. Pg 5 of the SI material states that aaRS enzymes were added to 5 μM. What enzymes were used? Did they have different activities with the noncanonical amino acids. Were any experiments done to determine if the failure of the CDPS enzymes with some substrate combinations was in fact a failure of the aaRS to load the tRNA?

Page 4. Bottom paragraph. SI tables 1 and 2 use the heading "present" for the ability of the enzymes to produce the alternate CDPs. Were these libraries of substrates incubated in a single complex reaction or tested individually. What level of detection warranted inclusion as present?

Page 5. Top paragraph introduces the PheRS and in particular the A294G mutant. Is this different than the aaRS used previously? How was it determined that the unusual AAs were indeed recognized by the

PheRS mutant. Additionally, the methods, the text, the Figure 2 legend, and SI Tables 1 and 2 are inadequate to determine which enzymes, which aaRS are used and how the different CDPS substrates are built. I realize that the authors wish to focus on the loaded tRNA pool (here) and the CDPS promiscuity (later) however the method is not suitably described to understand how the different experiments were performed.

Page 6, paragraph 2. Again, the use of "prior to this work" is unclear. Are the authors saying that prior to their publication of Ref 20, combinations of aa-tRNA and aa-DBE have not been used, or prior to the current manuscript?

Page 6, final paragraph. The novel trapped acyl-enzyme assay needs to be described more completely. Lacking context provided about the P1/P2 pockets, it is not at all clear why Figure 3C shows that ParaCDPS binds Phe in the P1 pocket.

The legend for Figure 3 needs to be re-written. The legend for Figure 3b should state that ParaCDPS is shown on the left (in purple) and ParcuCDPS on right (in orange). The legend for Figure 3d does not tell us which enzymes are used. The inset is undefined. While lines were fit to determine a rate, rate values are not actually presented nor discussed.

Page 9, top paragraph. The authors now introduce the XYP subfamily of CPDSs without context. This subfamily has not been defined, nor contrasted with the NYH subfamily to which ParcuCDPS and ParaCDPS (perhaps?) belong.

Page 10, top paragraph. Is it more plausible that the positive charge of the P2 pocket is more important for binding tRNA than for binding the glutamate side chain of an alternate substrate?

Page 10, bottom paragraph. Again, please clarify the use of "now". Are successful efforts to modify CDPS selectivity described in ref 10, or are the authors stating that their current efforts reflect the first successful changing of a CDPS. Are the similarities between HisRS and the P1 pocket really sufficient to guide the authors efforts. Or do the prior crystal structures of CDPS enzymes bound to substrates define the P1 pocket to support their mutagenesis efforts.

Figure 5. Again, please clarify identity and source of aaRS enzymes. Also clarify which tRNA source was used here.

The legend for Figure S1 should state that all mutants were of ParcuCDPS and not ParaCDPS.

Reviewer #2 (Remarks to the Author):

Herein, Sutherland and coworkers describe the characterization and expansion of substrate tolerance for model CDPSs that catalyze cyclodipeptide formation from His-tRNA substrates. Their work includes in vitro assembly of new cyclic dipeptides from tRNAs charged with unnatural amino acid analogs, the X-ray crystal structure of a model CDPS that catalyzes formation of His-containing cyclic dipeptides, and the directed evolution of this CDPS to redefine its substrate scope. Taken together, these results significantly expand understanding of the CDPS family.

There are several points that must be clarified or tested prior to this work potentially becoming suitable for publication:

—Whereever stereochemistry is known, it should be designated in the text. For example, on page 1, cyclo(His-Phe) should be revised to cyclo(L-His-L-Phe) if the configuration has been previously confirmed.

—Negative control data (e.g. using boiled enzyme and/or missing substrate) appear to be missing for enzyme activity/product formation assays throughout the mansucript. Examples include Fig. 1c and SI Fig. 6.

—On page 3, the authors indicate that they "produced both enzymes in high yield and purity and confirmed their activity and products using purified components in vitro (SI Fig. 1)." Based on this wording, I anticipated SI Fig. 1 to show evidence for the biosynthetic DKP products, but instead it shows SDS-PAGE of purified wild-type and mutant proteins. Revise this sentence to clarify what is actually included in SI Fig 1, and the in vitro enzyme assay data should also be added in SI.

—Fig 2b shows yields of cHF and cHP from multiple tRNA sources (i.e. S30 extract and E coli pool extraction). The text (page 4) indicates that they also performed reactions with in vitro transcribed tRNA. For comparison, these data should also be included in Fig. 2b to further validate their claim that E. coli pool is superior to the other tRNA generation methods.

—On page 4, the authors mention that "Hartman et al. previously determined the ability of aaRSs to accept non-canonical amino acids…." Since a substantial number of prior works have shown that aaRSs accept ncAAs, what is the rationale for only mentioning Hartman by name? Since this is a broad and well-established field of research, it would be more appropriate to include a reference to one of the review articles on aaRSs/ncAAs.

—Compared to the in vivo system developed by Canu et al. (ref 27), Sutherland et al.'s system for DKP generation from ncAAs is unique in that it was conducted in vitro. This is important and worth mentioning/highlighting, but seems buried in the manuscript.

—At bottom of page 4, reader is directed to SI Table 1-2 for summary of all analogs tested. Where are the LCMS data showing formation (or lack thereof) of DKPs from all amino acid analogues tested? This information should also be provided for all analogues.

—For Fig 5b, the data represented in bar graph should be clarified. Specifically, do these bars show the average cHX as %WT? If so, what was the number of replicates? Error bars should be included if possible and appropriate.

Reviewer #3 (Remarks to the Author):

In "Active site remodelling of a cyclodipeptide synthase redefines substrate scope", Sutherland et al study and modify substrate preferences of cyclodipeptide synthase enzymes. They first produce libraries of CDPs in vitro by supplying various non-canonical amino acids to CPDSs, tRNA pool, and aminoacyl synthetases. Next, they use small molecule analogs to investigate which site was bound by which substrate. Structures of ParcuCDPS were determined and mutants were made to analyze contribution to catalysis and to analyze and expand substrate scope.

Overall, this is a nice study that adds to the cyclodipeptide synthase field. The biochemistry and crystallography appear well performed, although the .cif files provided seem to be defective and cannot be opennbed with Pymol or Chimera.

The study should be publishable after minor corrections.

P4, top: Please add a phrase describing basis of the Mechulam et al method here so the casual reader understand the idea without needing look at the supplemental methods. Something like "Using this method, which is based on organic extraction of total tRNA, a highly…"

Fig 1 is odd. It seems more like a graphical abstract than a main figure. Panel B especially – this seems to be a summary of results presented in later figures.

Fig 2c: The light blue / dark blue and light purple / dark purple contrast is not quite distinct enough to clearly denote canonical vs non-canonical amino acid residues easily. Please modify.

P9, top, "The structure was solved by Iodide SAD phasing after extensive failed trials of molecular replacement, suggesting significant deviation from previously determined CDPS structures.":
-Change "suggesting significant deviation" to "suggesting substantial deviation", as no statistics are available.
-Please show a superimposition of ParcuCDPS with most similar known CDPS in the supplemental material to complement Fig 4b.

Page 10: "Therefore, the disruption of activity is caused exclusively by the change in these few residues which appear important for the enzyme to produce a CDP.": How do the authors explain the large shift in melting temperature shown in Fig S10?

The authors have solved many structures of mutants and show very little data about these structures, other than a backbone overlay. A couple more sentences would be welcome.
Supplemental table 3: Why are there only 2 water molecules for 7QAT, while there >40 for 7QB8? The resolutions of the two structures are pretty similar.

Supplemental: "Horse hear myoglobin" missing a "t", and "Horse" shouldn't be capitalized

Response to reviewers

We thank the reviewers for raising important points which strengthen the manuscript. Points raised by reviewers are shown in bullet points, response to comments are in red, and changes to text are in blue. All changes are marked in the manuscript.

Reviewer #1

- The structure determination seems well done with one exception. The diffraction statistics all look quite good. The crystallographic R-factors however all are above 20%. Looking at the number of solvent molecules, which range from 2 to 40 molecules, this may be the cause of the unusually high R-factors. For 1.9Å – 2.3 Å datasets, the authors may wish to see if their density allows for the inclusion of more waters, which may allow the refinements to proceed to completion.

We thank the reviewer for their comments and have refined the structures again, which have brought down the R-factors slightly. Although above 20%, the R-factors are consistently around 25% for all structures, which is acceptable and similar to other deposited structures at equivalent resolution. Some additional water molecules have been added to the structures where there was electron density suggesting their presence. We collected a new dataset for the E174L mutant, which did not suffer from excessive ice-rings. The refinement statistics for the new E174L mutant structure are a considerable improvement. The crystallisation table (Supplementary Table 3) now has the newly refined statistics.

- The biochemical experiments are well done. As noted below, the description of experimental methods and rationale for experiments is often minimal and the authors must revise the text to make sure that they are unambiguous or provide sufficient detail to analyze or reproduce these experiments.

We thank the reviewer for highlighting this, and we added details as specified below.

- Additionally, a few sentences in the Results section that better place the experiments in context will save the reader from needing to read the methods section to understand the experiments.
  We thank the reviewer for highlighting this and added some sentences to put experiments in context. Some examples:
  Page 5: “Consequently we explored the capability of *Para*CDPS and *Parcu*CDPS in accepting unnatural amino acid analogues within our *in vitro* experimental setup as described previously”
  Other examples where we think context was present:
  Page 9: “To test if hybrid reaction conditions would yield product,”

Page 10: “Following these experiments we focused on understanding histidine selection, and more specifically on P1, as it was predicted to possess a narrower binding pocket. To do this, we solved the crystal structure”

- Finally, some journals have policies about statements of priority (“first example of ...” page 13) and the authors may wish to soften some of their pronouncements that “their work pioneers...” or about their “remarkable shift in substrate specificity” or “diverse library of unprecedented compounds.”

We thank the reviewer for this comment and have added context to our findings, as well as rephrased instances where such references were made. For example

Abstract: “Here, we developed an *in vitro* system to generate” and “Our work successfully engineered a CDPS”

Fig1-legend: “**b**, strategy employed here, using a single enzyme catalyst to produce several cyclodipeptide products;”

Page 7: “Previously, the use of a shortened tRNA was employed in combination with aatRNAs on the CDPS from *Nocardia brasiliensis*[1], but the hybrid reaction conditions detailed herein use a unique combination of aa-DBE and aatRNA as substrates which has not previously been published.”

Page 15: “The products of the *Parcu*CDPS variants with changed specificity – cLP and cFP”

Page 15” This *in vitro* method has many advantages such as reaction scalability and has proven”

- Page 1. Paragraph 1 of introduction. The final two sentences are unclear about whether “this work” refers to references 8-10 or refers to the current manuscript.

The sentence “Prior to this work..” has been changed to “Prior to the work reported herein,...”

- Page 2. Bottom paragraph. The paper would benefit from an introduction to the mechanism of the CDPS enzymes that more fully describes the P1 and P2 pockets and the acyl-enzyme intermediate. The top of page 7 is the first place in which the authors note that the reaction proceeds through acylation. The roles of the specific pockets are never fully elaborated, only the broader differences of P1 being deeper and more restrictive. Similarly, it seems an oversight to not more clearly describe the existing structure of CDPS enzymes, in particular the AlbC acyl enzyme intermediate, which would place the current effort in proper context.

This is a very good suggestion, and a paragraph was added to the introduction to give the reader more context on previous information known about CDPSs including their mechanism of action, the two structural sub-groups

and the pockets they use for product formation. This paragraph begins on page 1: "AlbC from *Streptomyces noursei* was the first CDPS to be discovered in 2002.[2] Since then, there have been 120 CDPSs with empirically determined function and more than 1000 predicted members of the family (Uniprot: IPRO38622). CDPSs are known for their promiscuity as most are capable of naturally synthesising more than one product.[3] This interesting characteristic is due to two separate solvent accessible pockets (referred to as P1 and P2) which interact with the specific aminoacylated tRNA (aatRNA) required for product formation.[4] Extensive investigation into the mechanism using both experimental and computational methods elucidated the catalytic mechanism of CDPSs.[5, 6] The reaction proceeds via a ping pong reaction, the first half reaction in P1 forms an aminoacyl-enzyme intermediate covalently bound to the catalytic serine (S26 in AlbC).[7] The second step forms a dipeptidyl-enzyme intermediate which undergoes cyclisation facilitated by a conserved tyrosine residue. [6] This reaction relies heavily on four known residues which are conserved throughout the family: S26, Y167, E171 and Y189 (numbering respective to AlbC). Further research into CDPSs has highlighted that the family could be characterised into two sub-groups based on the residues present on P1/P2. The larger of the two families, NYH, features N40, Y202 and H203 whilst XYP has X40 where X is non-conserved, Y202 and P203.[8] Crystal structures from representatives of both families are available: AlbC; Rv2275; YvmC,[9] and BtCDPS[10] from NYH sub-family and *Nbra*-CDPS, *Rgry*-CDPS, *Fdum*-CDPS from XYP.[8] "

We also added a panel to figure 1 with the current proposed mechanism for the reaction catalysed by CDPS enzymes.

- Page 4. Top paragraph. The last paragraph of Page 3 states that the authors wished to avoid purification of individual AA-tRNA synthetases. The paragraph on page 4 then says that they performed their assay with the tRNA pool and the aaRS of choice. No method for purification of an aaRS is provided. Please clarify the source of the aaRS enzymes. Pg 5 of the SI material states that aaRS enzymes were added to 5 µM. What enzymes were used?

We thank the reviewer for pointing this out. Five aaRS were purified in our lab following published protocols from the literature. The methods (Page 5 of the SI) have been updated to include the purification of the aaRSs used in the experiments described in our manuscript.

- Did they have different activities with the noncanonical amino acids. Were any experiments done to determine if the failure of the CDPS enzymes with some substrate combinations was in fact a failure of the aaRS to load the tRNA?

We thank the reviewer for raising this point. All analogues tested by us with the exception of 4-bromo-L-proline, 4-amino-L-proline and 4-amino-L-phenylalanine have been previously demonstrated to be substrates for *E. coli* aaRS enzymes by several research groups. Reference 33 in the manuscript Hartman, M. C. T. Non-canonical Amino Acid Substrates of E. coli Aminoacyl-tRNA Synthetases. *ChemBioChem* **22**, 1-17, doi:https://doi.org/10.1002/cbic.202100299 (2021) recently reviewed all the non-canonical amino acids showed to act as substrates for aaRS. Cyclodipeptide synthases have been used as a coupled assay for aaRS function[11], since aminoacylation assays available for directly monitoring amino acid transfer are discontinuous and rely on radioactivity labelling[12] or electrophoretic mobility involving several steps [13]. A direct protocol using LC-MS has been used to monitor tRNA modifications (but not aminoacylation)[14]. Aiming to address this point raised by the reviewer we performed this assay but our results showed poor signal to noise in the positive control (aminoacylation with histidine) using tRNA pool and purified tRNA. We then conducted the same assay using a series of analogues and did not see peaks corresponding to the adenosine-amino acid adduct expected. Due to the noise intrinsic to the assay and the fact that non canonical amino acids would likely show lower levels of aminoacylation we cannot conclusively state whether aminoacylation took place or not. We added the graphs here so the reviewer can evaluate the noise in the experiment but have changed the manuscript to clarify that we cannot rule out aaRS selecting against some of the analogues used, particularly the 3 that were not previously demonstrated to be substrates for aaRS.

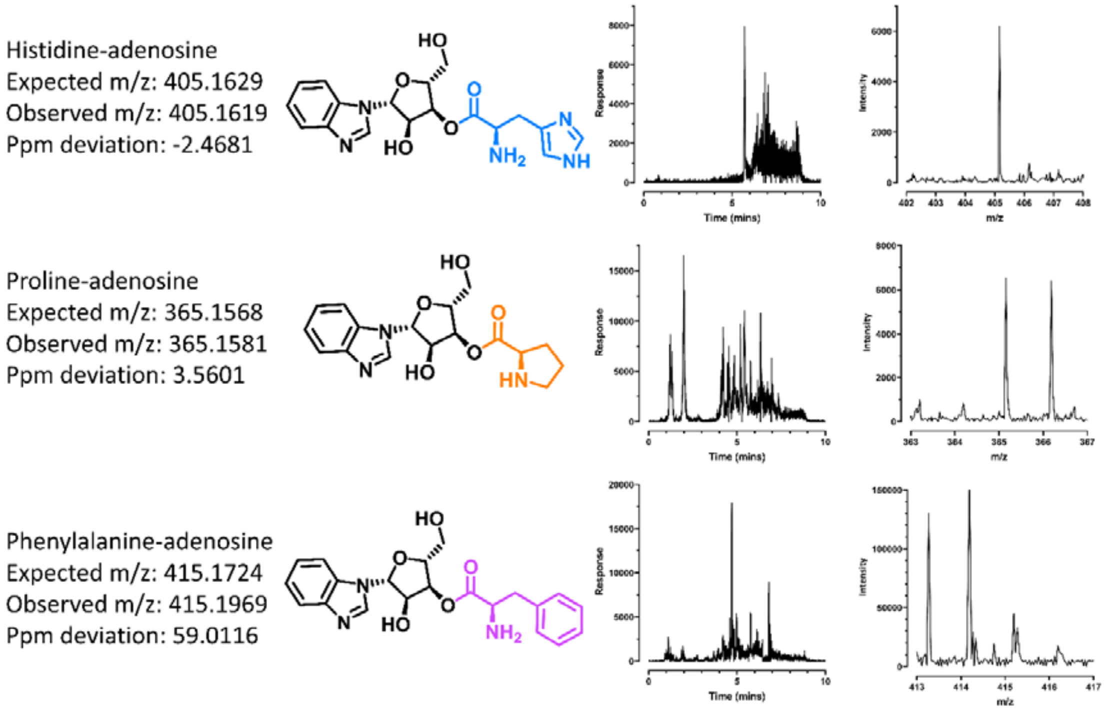

Change can be seen in this sentence on page 5:

"Since all analogues employed were previously shown to be substrates for histidinyl tRNA synthetase (HisRS)[15], the CDPS enzyme could be posing an additional sieve for substrate selection."

And on page 6:

"All but two of the known non-canonical amino acids previously shown to be accepted by PheRS-A294G"

- Page 4. Bottom paragraph. SI tables 1 and 2 use the heading "present" for the ability of the enzymes to produce the alternate CDPs. Were these libraries of substrates incubated in a single complex reaction or tested individually.

The wording has been changed to reflect that the unnatural amino acid substrates were tested individually: "All non-canonical amino acids tested with ParcuCDPS in individual assays are listed here and rows in green highlight the accepted substrates"

- What level of detection warranted inclusion as present?

Sentences were added on page 5 to explain the threshold employed to resolve if an unnatural amino acid has been incorporated into the CDP ring: "We imposed strict allowances on the mass deviation to confidently assess if an unnatural amino acid was accepted. All experiments were carried out using a high resolution mass spectrometer so only unique peaks (in comparison to controls) with area >100 and observed masses under 5 ppm from the predicted exact mass were taken into consideration for a product to be deemed 'present'."

- Page 5. Top paragraph introduces the PheRS and in particular the A294G mutant. Is this different than the aaRS used previously? How was it determined that the unusual AAs were indeed recognized by the PheRS mutant.

All analogues tested with the exception of 4-amino-L-phenylalanine have been previously shown to be substrates for PheRS-A294G, references 33 and 36 of the paper (below) further detail this aspect. All assays requiring $^{Phe}tRNA_{Phe}$ have used this mutant in our manuscript, and we clarified this point on SI page 6: "All assays requiring aminoacylation of $tRNA_{Phe}$ used the mutant PheRS-A294G."

33 Hartman, M. C. T. Non-canonical Amino Acid Substrates of E. coli Aminoacyl-tRNA Synthetases. *ChemBioChem* **22**, 1-17, doi:https://doi.org/10.1002/cbic.202100299 (2021).

36 Kast, P. & Hennecke, H. Amino acid substrate specificity of Escherichia coli phenylalanyl-tRNA synthetase altered by distinct mutations. *Journal of Molecular Biology* **222**, 99-124, doi:https://doi.org/10.1016/0022-2836(91)90740-W (1991).

- Additionally, the methods, the text, the Figure 2 legend, and SI Tables 1 and 2 are inadequate to determine which enzymes, which aaRS are used and how the different CDPS substrates are built. I realize that the authors wish to focus on the loaded tRNA pool (here) and the CDPS promiscuity (later) however the method is not suitably described to understand how the different experiments were performed.

We thank the reviewer for pointing this out and have added additional tables and text to the methods in the SI (page 6-7) detailing all the components and final concentrations used in each enzyme activity assay – tRNA pool, S30 extract, DBE minimal substrate.

"All assays requiring aminoacylation of $tRNA_{Phe}$ used the mutant PheRS-A294G. When *Parcu*CDPS was used, amino acid analogues of histidine and proline were in employed, in combination with ProRS and HisRS. When *Para*CDPS was used, amino acid analogues of histidine and phenylalanine were in employed, in combination with PheRS-A294G and HisRS. Only one pair of amino acid substrates was used at a time, so that only one substrate could be occupying P1 or P2."

General description of assay conditions using tRNA pool:

**Component**	**Final Concentration**
HEPES pH 7.0	100 mM
KCl	100 mM
$MgCl_2$	10 mM
ATP	5 mM
DTT	10 mM
Amino acid 1	500 µM
Amino acid 2	500 µM
tRNA pool	50 µM
aa-tRNA synthetase 1	5 µM
aa-tRNA synthetase 2	5 µM
CDPS	5 µM

General description of assay conditions using S30 extract:

**Component**	**Final Concentration**
HEPES-KOH pH 8.0	55 mM
Magnesium acetate	14 mM
Potassium acetate	50 mM
Ammonium acetate	155 mM
ATP	5 mM
Amino acid 1	2.5 mM
Amino acid 2	2.5 mM
S30 extract	22 mg/mL

CDPS	5 µM

- Page 6, paragraph 2. Again, the use of "prior to this work" is unclear. Are the authors saying that prior to their publication of Ref 20, combinations of aa-tRNA and aa-DBE have not been used, or prior to the current manuscript?

We agree this sentence is unclear as it reads and have changed it to : "the hybrid reaction conditions detailed herein use a unique combination of aa-DBE and aatRNA as substrates which has not previously been published."

- Page 6, final paragraph. The novel trapped acyl-enzyme assay needs to be described more completely. Lacking context provided about the P1/P2 pockets, it is not at all clear why Figure 3C shows that ParaCDPS binds Phe in the P1 pocket.

Information about the P1/P2 pockets has been added to the introduction (page 2) for better contextualisation of the results shown here. An additional cartoon scheme was added to panel 3C to clarify the reaction shown by the mass spectrometry.

- The legend for Figure 3 needs to be re-written. The legend for Figure 3b should state that ParaCDPS is shown on the left (in purple) and ParcuCDPS on right (in orange).

The figure legend for 3b was updated to: "b. quantification of product yield from aa-DBE and aatRNA reactions with each CDPS. Reactions with *Para*CDPS are shown in purple on the left and reactions with *Parcu*CDPS are in orange on the right. "

- The legend for Figure 3d does not tell us which enzymes are used. The inset is undefined. While lines were fit to determine a rate, rate values are not actually presented nor discussed.

We thank the reviewer for pointing this out. Figure legend updated to "time course assay for product formation of cHP and cHF using ParcuCDPS and ParaCDPS respectively. Lines are fit to an exponential equation to obtain rates of formation as follows: His-tRNA + Phe-tRNA = 0.0024 min^{-1}, His-tRNA + Phe-DBE = 0.0048 min^{-1}, His-DBE + Phe-tRNA = 0.0022 min^{-1}, His-DBE + Phe-DBE = too low to accurately fit, His-tRNA + Pro-tRNA = 0.05 min^{-1}, His-DBE + Pro-tRNA = 0.00002 min^{-1}, His-tRNA + Pro-DBE and His-DBE + Pro-DBE were too low to accurately fit. Inset shows that a very small of product is formed when one aa-DBE substrate is used, but not in the reaction with two aa-DBE substrates."

- Page 9, top paragraph. The authors now introduce the XYP subfamily of CPDSs without context. This subfamily has not been defined, nor contrasted with the NYH subfamily to which ParcuCDPS and ParaCDPS (perhaps?) belong.

This information has been added to the introduction where we discuss the two sub-groups NYH and XYP and how CDPS enzymes can be characterised into either group (Page 2): "This reaction relies heavily on four known residues which are conserved throughout the family: S26, Y167, E171 and Y189 (numbering respective to AlbC). Further research into CDPSs has highlighted that the family could be characterised into two sub-groups based on the residues present on P1/P2. The larger of the two families, NYH, features N40, Y202 and H203 whilst XYP has X40 where X is non-conserved, Y202 and P203.[8] Crystal structures from representatives of both families are available: AlbC; Rv2275; YvmC,[9] and BtCDPS[10] from NYH sub-family and *Nbra*-CDPS, *Rgry*-CDPS, *Fdum*-CDPS from XYP.[8]"

- Page 10, top paragraph. Is it more plausible that the positive charge of the P2 pocket is more important for binding tRNA than for binding the glutamate side chain of an alternate substrate?

This is a good observation and could be possible that the tRNA body is interacting with the positive charges on P2. A low resolution (5 A) structure published of an aatRNA-CDPS complex suggests a different stretch of positive residues (not on P2) participates in interactions with the tRNA. Because Parcu-CDPS has a structure that diverges from the one in this complex, interactions with aatRNA could occur. To illustrate this we added the sentence below on page 13:

"Interactions with negatively charged groups in the tRNA are also possible. Although a low resolution complex structure between $^{Phe}tRNA_{Phe}$ and the CDPS from *Candidatus Glomeribacter gigasporarum* indicates no such interactions occur on P2[16], the structure of Parcu-CDPS is significantly divergent and a different tRNA binding orientation could occur."

- Page 10, bottom paragraph. Again, please clarify the use of "now". Are successful efforts to modify CDPS selectivity described in ref 10, or are the authors stating that their current efforts reflect the first successful changing of a CDPS.

This is an important point as no prior attempt has been successful on altering the substrate specificity of a CDPS enzyme. Reference 10 discusses this and cites other works that attempted to change specificity without success, stating "CDPSs: attractive but tricky candidates for enzyme engineering… Several studies based on this rational engineering approach have shown that the substitution of one or two P1 or P2 residues can influence cyclodipeptide yields, but these studies were unable to generate novel specificities."

To make this point clearer, we rephrased the sentence to say:

"However, altering the substrate scope of a CDPS by mutating select residues has been unsuccessful (recently reviewed on **[17]**)."

- Are the similarities between HisRS and the P1 pocket really sufficient to guide the authors efforts. Or do the prior crystal structures of CDPS enzymes bound to substrates define the P1 pocket to support their mutagenesis efforts.

Prior structures showed a mostly uncharged P1 lined with leucine, valine, and phenylalanine residues. Therefore, determinant factors for amino acid recognition were difficult to pinpoint, and although the structure of AlbC covalently bound to a diphenylalanine peptide intermediate allows inferences to be made about substrate positioning, it differs from Parcu-CDPS to a large extent. The structure of Parcu-CDPS revealed a pocket lined with residues that could take part in hydrogen bonding interactions, and we hypothesized they were important for specific histidine recognition, akin to HisRS. We changed this sentence to reflect these two factors: "Inspired by HisRS and histidine recognition more generally (Supplementary Figure 11), and by the only high resolution structure of a CDPS with a ligand bound[18]"

- Figure 5. Again, please clarify identity and source of aaRS enzymes. Also clarify which tRNA source was used here.

Sentence included in the text (page 11) to confirm the aaRS and tRNA used: "This assay involved the use of the purified tRNA pool in conjunction with the amino acid tRNA synthetases (HisRS and ProRS) to yield cHP."

Additionally, we changed the legend of figure 5b to highlight which tRNA and aaRS were used in this reaction "cHP activity assay for reach set of mutants using the tRNA pool and purified aaRSs mentioned previously."

- The legend for Figure S1 should state that all mutants were of ParcuCDPS and not ParaCDPS.

"**Supplementary Figure 2.** Intact protein mass spectrometry analysis of *Parcu*CDPS variants used to confirm the desired mutation was present." Was added to figure legend of S1.

Reviewer #2 (Remarks to the Author):

Herein, Sutherland and coworkers describe the characterization and expansion of substrate tolerance for model CDPSs that catalyze cyclodipeptide formation from His-tRNA substrates. Their work includes in vitro assembly of new cyclic dipeptides from tRNAs charged with unnatural amino acid analogs, the X-ray crystal structure of a model CDPS that catalyzes formation of His-containing cyclic dipeptides, and the directed evolution of this CDPS to redefine its substrate scope. **Taken together, these results significantly expand understanding of the CDPS family.**

There are several points that must be clarified or tested prior to this work potentially becoming suitable for publication:

- Whereever stereochemistry is known, it should be designated in the text. For example, on page 1, cyclo(His-Phe) should be revised to cyclo(L-His-L-Phe) if the configuration has been previously confirmed.

We thank the reviewer for this comment, and all amino acid names have been updated to include known stereochemistry and the structures of cyclic dipeptide products have been changed to show the configuration.

- Negative control data (e.g. using boiled enzyme and/or missing substrate) appear to be missing for enzyme activity/product formation assays throughout the mansucript. Examples include Fig. 1c and SI Fig. 6.
  We thank the reviewer for pointing this out. All LC-MS experiments always had controls that omitted tRNA or enzymes (CDPS and/or aaRS) To make this clear we added a panel to Figure 1d showing the control and we have added Supporting Figures 9 and 10 which show negative results. Figure 1d legend now reads: "d, representative LC-HRMS spectra showing production of new molecules. The extracted ion chromatogram (EIC) for each is shown with a control EIC as an inset alongside the corresponding mass spectra from each peak. Spectra for all cyclodipeptides described here are on SI Figure 7 and Figure 8."

- On page 3, the authors indicate that they "produced both enzymes in high yield and purity and confirmed their activity and products using purified components in vitro (SI Fig. 1)." Based on this wording, I anticipated SI Fig. 1 to show evidence for the biosynthetic DKP products, but instead it shows SDS-PAGE of purified wild-type and mutant proteins. Revise this sentence to clarify what is actually included in SI Fig 1, and the in vitro enzyme assay data should also be added in SI.

We agree the sentence reads confusing, and therefore modified it to "We produced both enzymes in high yield, purity confirmed by intact protein mass spectrometry and SDS-PAGE analysis and their activity and products were confirmed using purified components *in vitro* (Supplementary Figure 1)."

- Fig 2b shows yields of cHF and cHP from multiple tRNA sources (i.e. S30 extract and E coli pool extraction). The text (page 4) indicates that they also performed reactions with in vitro transcribed tRNA. For comparison, these data should also be included in Fig. 2b to further validate their claim that E. coli pool is superior to the other tRNA generation methods.

We thank the reviewer for this suggestion and have now added the IVT reaction results to Figure 2b.

- On page 4, the authors mention that "Hartman et al. previously determined the ability of aaRSs to accept non-canonical amino acids…." Since a substantial number of prior works have shown that aaRSs accept ncAAs, what is the

rationale for only mentioning Hartman by name? Since this is a broad and well-established field of research, it would be more appropriate to include a reference to one of the review articles on aaRSs/ncAAs.

We thank the reviewer for this comment, and we rephrased the sentence as follows "Recently, non-canonical amino acid acceptance by aminoacyl-tRNA synthetases from *E.coli* was reviewed, summarizing extensive research in this field by several labs worldwide.[19] The use of non-canonical substrates with CDPS was previously carried out employing *in vivo* experiments exclusively.[20]"

- Compared to the in vivo system developed by Canu et al. (ref 27), Sutherland et al.'s system for DKP generation from ncAAs is unique in that it was conducted in vitro. This is important and worth mentioning/highlighting, but seems buried in the manuscript.

This finding has been incorporated in the text from the abstract (page 1: "Here, we developed an *in vitro* system to generate..") to results (page 5: "Consequently we explored the capability of *Para*CDPS and *Parcu*CDPS in accepting unnatural amino acid analogues within our *in vitro* experimental setup as described previously.") to the conclusion (page 15: "This *in vitro* method has many advantages such as reaction scalability and has proven useful..") to further highlight this to the reader.

- At bottom of page 4, reader is directed to SI Table 1-2 for summary of all analogs tested. Where are the LCMS data showing formation (or lack thereof) of DKPs from all amino acid analogues tested? This information should also be provided for all analogues.

We thank the reviewer for highlighting this. Supplementary Figure 7 and Supplementary Figure 8 have the LC-MS data for the analogues that were produced, and we added data for the unsuccessful analogues as well on Supplementary Figures 9 and 10.

- For Fig 5b, the data represented in bar graph should be clarified. Specifically, do these bars show the average cHX as %WT? If so, what was the number of replicates? Error bars should be included if possible and appropriate.

The legend of Figure 5b currently states "The activity is shown as a percentage of the wild-type *Parcu*-CDPS activity.". We decided to report the data as a fraction of WT because some values are very small and therefore would be difficult to visualize on the same graph. For panel b errors were spread using the standard equation for error propagation in which fractional uncertainties add in quadrature so:

$$Q = \frac{parameter\ 1}{parameter\ 2}$$

$$\frac{proparagated\ error\ on\ Q}{Q} = \sqrt{\left({error\ on\ parameter\ 1}/{parameter\ 1}\right)^2 + \left({error\ on\ parameter\ 2}/{parameter\ 2}\right)^2}$$

All experiments for quantification were done in triplicate, and standard curves in duplicate, and all experiments, controls, standards were run on the same day with the same settings to ensure data would be robust. This is stated on the Supplementary information "To monitor the production of the cyclic dipeptide formed by the enzyme, a discontinuous time course of the activity assay was performed in triplicate.". We added this sentence to the figure legend: "Data is shown on panels b, c and d as the average + standard deviation of experiments conducted in triplicates where authentic commercial standards were used for calibration curves and quantification." We also updated the legend on Figure 5b to make clear errors were propagated:

"Errors were propagated accordingly to account for errors in each parameter individually."

Reviewer #3 (Remarks to the Author):

In "Active site remodelling of a cyclodipeptide synthase redefines substrate scope", Sutherland et al study and modify substrate preferences of cyclodipeptide synthase enzymes. They first produce libraries of CDPs in vitro by supplying various non-canonical amino acids to CPDSs, tRNA pool, and aminoacyl synthetases. Next, they use small molecule analogs to investigate which site was bound by which substrate. Structures of ParcuCDPS were determined and mutants were made to analyze contribution to catalysis and to analyze and expand substrate scope.

Overall, this is a nice study that adds to the cyclodipeptide synthase field. The biochemistry and crystallography appear well performed, although the .cif files provided seem to be defective and cannot be opennbed with Pymol or Chimera.

We apologize for this issue, as we are able to open the files. We have now provided the pdb files as well, as an additional way to open files.

The study should be publishable after minor corrections.

- P4, top: Please add a phrase describing basis of the Mechulam et al method here so the casual reader understand the idea without needing look at the supplemental methods. Something like "Using this method, which is based on organic extraction of total tRNA, a highly…"

We thank the reviewer for this comment and changed sentence to "We then adapted a procedure by Mechulam et al. which performs a liquid-liquid organic extraction of total tRNA, followed by subsequent clean up to produce a 'pool' of tRNA naturally found in E. coli."

- Fig 1 is odd. It seems more like a graphical abstract than a main figure. Panel B especially – this seems to be a summary of results presented in later figures.

We did this purposefully as Communications Chemistry does not have a separate graphical abstract figure. Therefore, if accepted manuscripts are depicted online as an abstract + Fig. 1, and we therefore thought a summary component to the figure would be worthwhile. We asked the editorial office if this was acceptable and their response is below, so we opted for leaving the figure largely unchanged, with the exception that a mechanism was added.

“I think you may be referring to the fact that a zoomed in version of Figure1 is displayed on the homepage akin to how graphical abstracts often appear. We don't allow submission of a graphical abstract so if you would like to prepare Fig 1 as a summary figure then I don't see a problem with that.”

- Fig 2c: The light blue / dark blue and light purple / dark purple contrast is not quite distinct enough to clearly denote canonical vs non-canonical amino acid residues easily. Please modify.

We changed colours of figures where chemical structures are present to comply with this valid suggestion.

- P9, top, “The structure was solved by Iodide SAD phasing after extensive failed trials of molecular replacement, suggesting significant deviation from previously determined CDPS structures.”: Change “suggesting significant deviation” to “suggesting substantial deviation”, as no statistics are available.

As suggested we changed the wording of this sentence to say “substantial deviation”.

- Please show a superimposition of ParcuCDPS with most similar known CDPS in the supplemental material to complement Fig 4b.

We thank the reviewer for this suggestion and have added a superimposition of *Parcu*CDPS and the most similar known CDPS (BtCDPS in this case). This figure can be found in the SI (page 22, Supplementary Figure 11).

- Page 10: “Therefore, the disruption of activity is caused exclusively by the change in these few residues which appear important for the enzyme to produce a CDP.”: How do the authors explain the large shift in melting temperature shown in Fig S10?

We understand the concern raised by reviewer, and repeated the DSF assay for all mutants purified (Figure S14A-C, page 28). Assays were done in triplicate as before and recapitulate the trend previously observed by which some mutants have larger Tm values. The wild type protein, for example, has one of the lowest Tm values, but despite this could be crystallized implying that it is not unfolded or particularly unstable. Reasons underlying this increase in Tm for several mutants are unclear, and we decided not to speculate.

- The authors have solved many structures of mutants and show very little data about these structures, other than a backbone overlay. A couple more sentences would be welcome. Supplemental table 3: Why are there only 2 water molecules for 7QAT while there >40 for 7QB8? The resolutiosn fo the two structures are pretty similar.

The dataset for E174L suffered from ice rings so we collected a new dataset, solved and refined the structure again, which has given a much better map and statistics. We have also added additional water molecules where there was density to do so and refined the structures again. We hope that working .cif files are now available so that you can see the structures and maps.

- Supplemental: "Horse hear myoglobin" missing a "t", and "Horse" shouldn't be capitalized. Fixed this error on page 9 of SI.

1. Canu, N., et al., *Flexizyme-aminoacylated shortened tRNAs demonstrate that only the aminoacylated acceptor arms of the two tRNA substrates are required for cyclodipeptide synthase activity.* Nucleic Acids Res, 2020. **48**(20): p. 11615-11625.
2. Lautru, S., et al., *The Albonoursin Gene Cluster of S. noursei: Biosynthesis of Diketopiperazine Metabolites Independent of Nonribosomal Peptide Synthetases.* Chem. Biol., 2002. **9**(12): p. 1355-1364.
3. Jacques, I.B., et al., *Analysis of 51 cyclodipeptide synthases reveals the basis for substrate specificity.* Nat Chem Biol, 2015. **11**(9): p. 721-7.
4. Moutiez, M., et al., *Unravelling the mechanism of non-ribosomal peptide synthesis by cyclodipeptide synthases.* Nat. Commun., 2014. **5**(1): p. 5141.
5. Sauguet, L., et al., *Cyclodipeptide synthases, a family of class-I aminoacyl-tRNA synthetase-like enzymes involved in non-ribosomal peptide synthesis.* Nucleic Acids Res., 2011. **39**(10): p. 4475-4489.
6. Schmitt, E., et al., *Cyclization Reaction Catalyzed by Cyclodipeptide Synthases Relies on a Conserved Tyrosine Residue.* Sci. Rep., 2018. **8**(1): p. 7031.
7. Vetting, M.W., S.S. Hegde, and J.S. Blanchard, *The structure and mechanism of the Mycobacterium tuberculosis cyclodityrosine synthetase.* Nat. Chem. Bio., 2010. **6**(11): p. 797-799.
8. Bourgeois, G., et al., *Structural basis for partition of the cyclodipeptide synthases into two subfamilies.* J. Struct. Biol., 2018. **203**: p. 17-26.
9. Bonnefond, L., et al., *Structural basis for nonribosomal peptide synthesis by an aminoacyl-tRNA synthetase paralog.* PNAS, 2011. **108**(10): p. 3912-3917.
10. Harding, C.J., et al., *Bypassing the requirement for aminoacyl-tRNA by a cyclodipeptide synthase enzyme.* RSC Chem. Bio., 2021. **2**: p. 230-240.
11. Grube, C.D. and H. Roy, *A continuous assay for monitoring the synthetic and proofreading activities of multiple aminoacyl-tRNA synthetases for high-throughput drug discovery.* RNA Biol, 2018. **15**(4-5): p. 659-666.

12. Ledoux, S. and O.C. Uhlenbeck, *[3'-32P]-labeling tRNA with nucleotidyltransferase for assaying aminoacylation and peptide bond formation.* Methods, 2008. **44**(2): p. 74-80.
13. Gamper, H. and Y.M. Hou, *A Label-Free Assay for Aminoacylation of tRNA.* Genes (Basel), 2020. **11**(10).
14. Salazar, J.C., et al., *A truncated aminoacyl-tRNA synthetase modifies RNA.* Proc Natl Acad Sci U S A, 2004. **101**(20): p. 7536-41.
15. Hartman, M.C.T., K. Josephson, and J.W. Szostak, *Enzymatic aminoacylation of tRNA with unnatural amino acids.* Proceedings of the National Academy of Sciences of the United States of America, 2006. **103**(12): p. 4356-4361.
16. Bourgeois, G., et al., *Structural basis of the interaction between cyclodipeptide synthases and aminoacylated tRNA substrates.* RNA, 2020. **26**(11): p. 1589-1602.
17. Canu, N., et al., *Cyclodipeptide synthases: a promising biotechnological tool for the synthesis of diverse 2,5-diketopiperazines.* Nat Prod Rep, 2020. **37**(3): p. 312-321.
18. Moutiez, M., et al., *Unravelling the mechanism of non-ribosomal peptide synthesis by cyclodipeptide synthases.* Nature Communications, 2014. **5**(1): p. 5141.
19. Hartman, M.C.T., *Non-canonical Amino Acid Substrates of E. coli Aminoacyl-tRNA Synthetases.* ChemBioChem, 2021. **22**: p. 1-17.
20. Canu, N., et al., *Incorporation of Non-canonical Amino Acids into 2,5-Diketopiperazines by Cyclodipeptide Synthases.* Angewandte Chemie International Edition, 2018. **57**(12): p. 3118-3122.

REVIEWERS' COMMENTS:

Reviewer #1 (Remarks to the Author):

The authors have addressed my prior concerns. The manuscript is clearer now and the introductory paragraph is particularly helpful.

Reviewer #2 (Remarks to the Author):

The authors have addressed my prior concerns, and the presentation style, rigor, and information content of this paper is quite strong. It will provide a significant contribution to the literature in this field.
